# Recent Advances in the Analytical Stress Field Solutions for Radiused Notches in Orthotropic Solids

**DOI:** 10.3390/ma16113915

**Published:** 2023-05-23

**Authors:** Alessandro Pontefisso, Matteo Pastrello, Michele Zappalorto

**Affiliations:** Department of Management and Engineering, University of Padova, Stradella San Nicola 3, 36100 Vicenza, Italy; alessandro.pontefisso@unipd.it (A.P.); matteo.pastrello@phd.unipd.it (M.P.)

**Keywords:** stress fields, analytical solutions, radiused notches, orthotropic solid

## Abstract

The main aim of this work is to provide a brief overview of the analytical solutions available to describe the in-plane and out-of-plane stress fields in orthotropic solids with radiused notches. To this end, initially, a brief summary on the bases of complex potentials for orthotropic elasticity is presented, with reference to plane stress or strain and antiplane shear problems. Subsequently, the attention is moved to the relevant expressions for the notch stress fields, considering elliptical holes, symmetric hyperbolic notches, parabolic notches (blunt cracks), and radiused V-notches. Eventually, examples of applications are presented, comparing the presented analytical solutions with the results from numerical analyses carried out on relevant cases.

## 1. Introduction

Accounting for the effects of geometrical variations is an essential step in the design process of a mechanical component. Stress raisers, indeed, may severely hamper the static and fatigue strength of mechanical parts, and designers are often required to accurately assess the local stress fields in the stress concentration regions, either numerically or analytically. 

Over the last 100 years and more, scientists and engineers devoted significant efforts to determining the stress fields around holes, notches, and cutouts, and the first steps in this direction can be dated back to the late 19th century or early 20th century [1,2,3,4]. Fundamental contributions in the field of linear elastic fracture and notch mechanics are those due to Williams [5], who described the stress field near sharp V-notches, and Irwin [6], who provided his renowned equation describing the stress fields near a sharp crack. 

Moving the attention to radiused notches, namely notches with a finite tip radius, worth mentioning is the paper by Creager and Paris [7] and the thorough work by Neuber [8], who provided the stress concentration factors for a large variety of notch problems. 

Many years later, Lazzarin and Tovo [9] provided a general expression for the mode 1 and 2 stress fields around blunt notches, demonstrating that Irwin, Williams, Creager and Paris, and Neuber’s solutions could be obtained as particular cases of their more general solution. From the previously-mentioned works, several analytical solutions have been developed and are available to designers for predicting the stress fields of components where different stress raisers are present [10,11,12]. 

A large variety of mode 3 notch problems was addressed by Zappalorto and co-workers (see among the others, [12,13,14] and references reported therein). 

All the above-mentioned solutions are valid for isotropic materials and, accordingly, cannot be used when dealing with materials characterized by an orthotropic or rectilinearly anisotropic elastic behavior, such as, for example, fiber-reinforced polymers, wood, or crystals.

Within the context of stress concentrations provoked by holes in bodies obeying an anisotropic elastic behavior, the contributions by Savin [15] and Lekhnitskii [16] are of fundamental importance and are usually regarded as milestones. Starting from the previously mentioned publications, Sih et al. [17] in 1965 provided the general equations for the stress fields at a crack tip in a rectilinearly anisotropic body making use of a complex variable approach, whereas the pointed V-notch case in an anisotropic plate was addressed, later, by many authors [18,19,20,21,22,23,24]. 

Ukadgaonker and Rao, instead, [25] provided the solutions for an orthotropic plate with triangular holes, and later extended the analysis to holes of irregular shapes [26,27].

Zappalorto and Carraro [28] proposed a theory for thick anisotropic plates weakened by sharp notches where the 3D governing equations of elasticity were reformulated to provide two uncoupled equations in the two-dimensional space. Hasebe [29] investigated the problem related to an orthotropic elastic half-plane weakened by an oblique edge-crack, and provided a general solution for an orthotropic elastic plane problem of an infinite plate weakened by a hole using a mapping function based on Lekhnitskii formalism [30].

Fil’shtyns’kyi et al. [31] targeted a specific application: the case of piezoceramic plates with parabolic or rectilinear cracks in the frame of magnetoelasticity. They studied the cross-dependencies of the SIF and the magnetic-induction intensity factor, providing the formulas for both.

Moving the attention to holes and radiused notches, worth a mention are the works by Bonora et al. [32,33], who reported a closed-form solution for composite laminates weakened by circular holes and subjected to in-plane stresses, whereas Chiang [34] addressed for the first time the plane problem of blunt cracks in anisotropic solids, providing an approximate solution. 

Wang [35] studied the case of boreholes drilled in the rock for petroleum engineering, deriving an analytical solution for the stress distribution around the borehole. The problem was regarded as a circular hole enveloped in an isotropic case, made of metal and surrounded by cement, embedded in an orthotropic matrix under plane strain conditions.

Hsieh and Wu [36] provided the full field solutions for an anisotropic elastic plate weakened by a hole, where its shape was obtained from the case of an elliptical hole by means of conformal mapping. In more detail, the authors used Stroh formalism and applied uniform loading at infinity, eventually claiming that their solutions are “the first verified correct full-field analytical solution published in the literature”.

Nguyen-Hoang and Becker [37] provided the solutions for the stress field in a composite orthotropic finite laminate under uniform tension weakened by an open-hole. They used complex potential formulations and validated the results with FE analyses. Eventually, they carried out a failure analysis by means of the Theory of Critical Distances and Finite Fracture Mechanics.

Khechai et al. [38] investigated stress concentrations in composite laminates weakened by holes under in-plane loadings. In particular, an extension of Greszczuk’s analytical solution for multilayer laminates was provided and validated with the Digital Image Correlation technique, considering several parameters such as fiber orientation, stacking sequence, type of load, and anisotropic ratio.

Huang et al. [39] studied the case of an anisotropic matrix containing an elliptic inhomogeneity with an interface that is imperfect, meaning an interface where normal or tangential displacements are discontinuous across the interface. In particular, the authors presented an explicit solution for the stresses in the case of remote plane tension and/or eigenstrain in the inhomogeneity by means of a complex series expansion, of which they determined the coefficients. By their approach, the solution of the stress fields in the case of perfect interface or debonded interface (i.e., hole) is obtained.

In 2016, Kazberuk et al. [40] presented the eigensolution of the quasi-orthotropic wedge problem, whilst Savruk and Kazberuk [41] presented the boundary value solution of sharp and rounded V-notches in a quasi-orthotropic plane. 

Chen [42] proposed an analytical approach for determining stress fields in the case of anisotropic plates weakened by a notch and subjected to bending. The solutions are based on Stroh formalism and account for the notch opening angle, material orientation, and magnitude of anisotropic behavior.

Savruk et al. [43] investigated the maximum stresses on the boundary of parabolic notches while varying the material orientation under the assumption of an infinite anisotropic plane. They provided the solutions for three different loading conditions: symmetric tension, transverse shear, and longitudinal shear.

The exact solution for anisotropic plates with blunt cracks and radiused slits was derived by Zappalorto and Carraro [44] using the Lekhnitskii approach. The same authors [45] later derived an approximate analytical solution for the two-dimensional stress distributions in orthotropic plates with blunt V-notches. An improved version of this last-mentioned solution is due to Pastrello et al. [46], whereas the extensions to mode 2 and mode 3 are due to Pastrello et al. [47] and Zappalorto and Salviato [48], respectively. 

The main aim of the present work is to present a brief overview of the above-mentioned analytical solutions, also providing some examples of application and a discussion on their accuracy degree. In particular, the paper presents the following structure:

- In Section 2, the fundamentals of complex potentials for orthotropic elasticity are discussed, with reference to plane stress or strain and antiplane shear problems.
- Section 3Sections toSection 6 present the analytical expressions for the mode 1, 2, and 3 stress fields related to different notch geometries, i.e., elliptical hole (Section 3), hyperbolic lateral notches (Section 4), parabolic notches, i.e., blunt cracks, (Section 5), and lateral radiused V-notches (Section 6).
- Eventually, Section 7 reports some examples of application, comparing the presented analytical solutions to the results from numerical analyses related to relevant cases.

## 2. Fundamentals of Complex Potential for Orthotropic Elasticity 

### 2.1. Plane Stress or Plane Strain Problems 

Consider a body obeying an orthotropic elastic behavior, according to which the elastic stress–strain relationships under plane stress can be formulated on the basis of four independent elastic constants:

(1) $$\left\{\begin{array}{c} \varepsilon_{x}\\ \varepsilon_{y}\\ \gamma_{\mathrm{xy}} \end{array}\right\}=\left[\begin{array}{ccc} S_{11} & S_{12} & 0\\ S_{12} & S_{22} & 0\\ 0 & 0 & S_{66} \end{array}\right]\left\{\begin{array}{c} \sigma_{x}\\ \sigma_{y}\\ \tau_{\mathrm{xy}} \end{array}\right\}$$

Stress fields in the considered orthotropic body can be written in terms of two complex functions as follows [16]:

(2) σxx=Reμ12φ1(z1)+μ22φ2(z2)σyy=Reφ1(z1)+φ2(z2)τxy=−Reμ1φ1(z1)+μ2φ2(z2)

or, equivalently, invoking stresses in polar coordinates:

(3) σrr=2Resinθ−μ1cosθ2φ1′(z1)+sinθ−μ2cosθ2φ2′(z2)σθθ=2Recosθ+μ1sinθ2φ1′(z1)+cosθ+μ2sinθ2φ2′(z2)τrθ=2Resinθ−μ1cosθcosθ+μ1sinθφ1′(z1)+sinθ−μ2cosθcosθ+μ2sinθφ2′(z2)

Under the condition that ${\left(2T_{12}+T_{66}\right)}^{2}\geq 4T_{11}T_{22}$, $\mu_{1,3}=\pm i\beta_{1}^{}$ and $\mu_{2,4}=\pm i\beta_{2}^{}$ are the conjugate roots of the following equation [27]:

(4) $$T_{11}\mu_{}^{4}+(2T_{12}+T_{66})\mu_{}^{2}+T_{22}=0$$

namely:

(5) $$\beta_{1,2}=\sqrt{\frac{2T_{12}+T_{66}\pm \sqrt{{\left(2T_{12}+T_{66}\right)}^{2}-4T_{11}T_{22}}}{2T_{11}}}$$

In Equations (4) and (5), T_ij_ equates the terms of the compliance matrix, S_ij_, for plane stress. In this case, invoking the engineering elastic constants, one obtains:

(6) $$\begin{array}{l} T_{11}=S_{11}=1/E_{x}\;\;T_{22}=S_{22}=1/E_{y}\;\;\\ T_{12}=S_{12}=-\nu_{xy}/E_{x}\;\;T_{66}=S_{66}=1/G_{xy} \end{array}$$

where E_x_ and E_y_ are the elastic moduli along the x and y directions, respectively, G_xy_ is the elastic modulus under shear, and ν_xy_ is the Poisson’s ratio. Accordingly, under plane stress conditions, Equation (7) can be re-written as:

(7) $$\beta_{1,2}=\sqrt{-\nu_{xy}+\frac{E_{x}}{2G_{xy}}\pm \sqrt{{\left(-\nu_{xy}+\frac{E_{x}}{2G_{xy}}\right)}^{2}-{\left(\frac{E_{x}}{E_{y}}\right)}^{}}}$$

Differently, in the case of plane strain conditions, T_ij_ equates B_ij_, defined as [28]:

(8) $$\begin{array}{l} T_{11}=B_{11}=\frac{S_{11}\;S_{33}-S_{13}^{2}}{S_{33}}\;\;T_{12}=B_{12}=\frac{S_{12}\;S_{33}-S_{13}\;S_{23}}{S_{33}}\\ T_{22}=B_{22}=\frac{S_{22}\;S_{33}-S_{23}^{2}}{S_{33}}\;\;\;\;T_{66}=B_{66}=S_{66}\;\; \end{array}$$

where $S_{33}=1/E_{z}$.

Equation (3) can also be conveniently re-written as [28]:

(9) σrr=2Rek11+i k12φ1′(z1)+k21+i k22φ2′(z2)σθθ=2Rem11+i m12φ1′(z1)+m21+i m22φ2′(z2)τrθ=2Ren11+i n12φ1′(z1)+n21+i n22φ2′(z2)

where the auxiliary angular functions introduced, k_ij_, m_ij_, and n_ij_, read as follows:

(10) $$k_{11}=\sin^{2}\theta -{\left(\beta_{1}\cos\theta \right)}^{2}\;\;\;\;\;k_{12}=-2\beta_{1}\sin\theta \cos\theta$$

(11) $$k_{21}=\sin^{2}\theta -{\left(\beta_{2}\cos\theta \right)}^{2}\;\;\;\;\;k_{22}=-2\beta_{2}\cos\theta \sin\theta$$

(12) $$m_{11}=\cos^{2}\theta -{\left(\beta_{1}\sin\theta \right)}^{2}\;\;\;\;\;k_{22}=-2\beta_{2}\cos\theta \sin\theta$$

(13) $$m_{21}=\cos^{2}\theta -{\left(\beta_{2}\sin\theta \right)}^{2}\;\;\;m_{22}=2\beta_{2}\sin\theta \cos\theta$$

(14) $$n_{11}=\frac{1}{2}\sin2\theta \left(1+\beta_{1}^{2}\right)\;\;\;\;\;n_{12}=-\beta_{1}\cos2\theta$$

(15) $$n_{21}=\frac{1}{2}\sin2\theta \left(1+\beta_{2}^{2}\right)\;\;\;\;\;n_{22}=-\beta_{2}\cos2\theta$$

### 2.2. Antiplane Shear Deformation Problems

Consider again a body obeying an orthotropic elastic behavior; under the hypothesis of pure antiplane deformation, the only non-vanishing stresses are τ_xz_ and τ_yz_, linked to the corresponding shear strains by the following relationships:

(16) $$\tau_{xz}=G_{xz}\gamma_{xz}\;\;\;\;\;\tau_{yz}=G_{yz}\gamma_{yz}$$

In the antiplane shear model, out-of-plane shear strains and stresses depend only on the x and y coordinates, so that the equilibrium and compatibility equations guarantee that the out-of-plane displacement, w, satisfies the following expression:

(17) $$G_{xz}\frac{\partial^{2}w}{\partial^{}x^{2}}+G_{yz}\frac{\partial^{2}w}{\partial^{}y^{2}}=0$$

The characteristic equation associated to Equation (17) is [16]:

(18) $$G_{yz}\mu_{3}^{2}+G_{xz}=0$$

with roots:

(19) $$\mu_{3}=\pm i\beta_{3}=\pm i\sqrt{\frac{G_{xz}}{G_{yz}}}$$

Under these conditions, the following expressions are valid for the out-of-plane shear stresses [28]:

(20) τzx=Reμ3φ3′(z3)     τzy=−Reφ3′(z3)

or in polar components:

(21) τzθ=−2Recosθ+i β3sinθφ3′(z3)τzr=2Re−sinθ+i β3cosθφ3′(z3)

where φ_3_ is a proper complex function to be chosen depending on the specific notch geometry under consideration.

## 3. Stress Fields for an Infinite Orthotropic Plate with an Elliptical Hole

### 3.1. Mode 1 Loadings 

**Figure 1:**
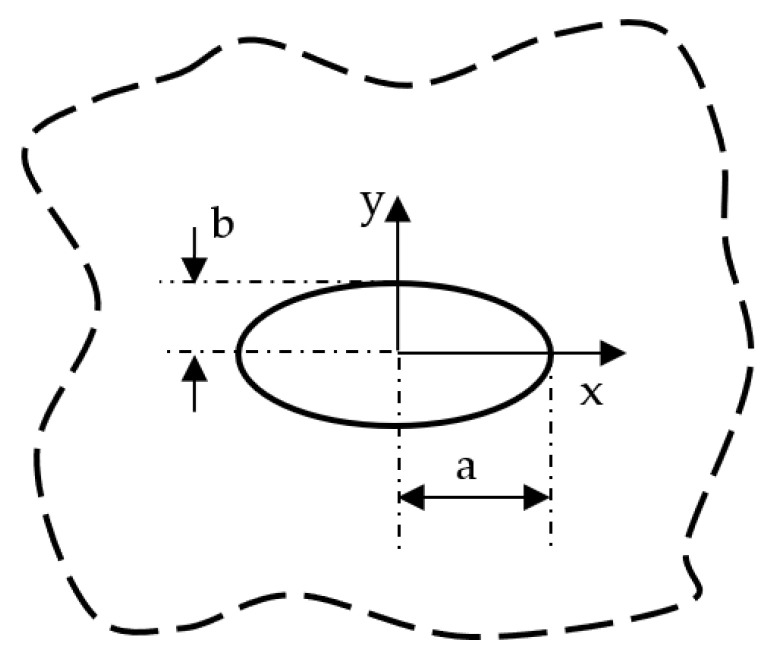
Elliptic hole in an infinite solid and reference system used for Equation (23).

Consider an infinite plate with an elliptical hole with major axis a and minor axis b (see Figure 1). The in-plane stress fields can be determined according to the solution proposed by Savin [15]. In particular, rearranging Savin’s solution, the mode 1 problem (far applied tension) can be tackled using the following complex potentials:

(22) $$\begin{array}{l} \varphi_{1}=\frac{\sigma_{yy}^{g}\beta_{2}}{2{\left(\beta_{1}-\beta_{2}\right)}^{2}}\left\{\frac{a\left(a+b\beta_{1}\right)\left(\beta_{1}-\beta_{2}\right)}{z_{1}+\sqrt{z_{1}{}^{2}-a^{2}+b^{2}\beta_{1}^{2}}}+z_{1}\beta_{2}\right\}\\ \varphi_{2}=\frac{-\sigma_{yy}^{g}\beta_{1}}{2{\left(\beta_{1}-\beta_{2}\right)}^{2}}\left\{\frac{a\left(a+b\beta_{2}\right)\left(\beta_{1}-\beta_{2}\right)}{z_{2}+\sqrt{z_{2}{}^{2}-a^{2}+b^{2}\beta_{2}^{2}}}+z_{2}\beta_{1}\right\} \end{array}$$

where z_1_ = x + μ_1_y, z_2_ = x + μ_2_y, and $\sigma_{yy}^{g}$ is the far applied tension in y direction. 

Substituting Equation (22) into Equation (2) results in the following expressions for the stress components:

(23) $$\begin{array}{c} \sigma_{\mathrm{xx}}=\frac{\sigma_{\mathrm{yy}}^{\mathrm{Tip}}b\beta_{1}\beta_{2}}{\left\{a\beta_{2}+\beta_{1}\left(a+b\beta_{2}\right)\right\}}\cdot \left\{\frac{a\left(a+b\beta_{1}\right)\beta_{2}\beta_{1}^{2}\Omega_{1}}{r_{1}\left(\beta_{1}-\beta_{2}\right)\Theta_{1}}-\frac{a\left(a+b\beta_{2}\right)\beta_{1}\beta_{2}^{2}\Omega_{2}}{r_{2}\left(\beta_{1}-\beta_{2}\right)\Theta_{2}}\right\}\\ \sigma_{\mathrm{yy}}=\frac{\sigma_{\mathrm{yy}}^{\mathrm{Tip}}b\beta_{1}\beta_{2}}{\left\{a\beta_{2}+\beta_{1}\left(a+b\beta_{2}\right)\right\}}\cdot \left\{1-\frac{a\left(a+b\beta_{1}\right)\beta_{2}\Omega_{1}}{r_{1}\left(\beta_{1}-\beta_{2}\right)\Theta_{1}}+\frac{a\left(a+b\beta_{2}\right)\beta_{1}\Omega_{2}}{r_{2}\left(\beta_{1}-\beta_{2}\right)\Theta_{2}}\right\}\\ \tau_{\mathrm{xy}}=\frac{\sigma_{\mathrm{yy}}^{\mathrm{Tip}}b\beta_{1}\beta_{2}}{\left\{a\beta_{2}+\beta_{1}\left(a+b\beta_{2}\right)\right\}}\cdot \left\{\frac{a\left(a+b\beta_{1}\right)\beta_{1}\beta_{2}\Lambda_{1}}{r_{1}\left(\beta_{1}-\beta_{2}\right)\Theta_{1}}-\frac{a\left(a+b\beta_{2}\right)\beta_{1}\beta_{2}\Lambda_{2}}{r_{2}\left(\beta_{1}-\beta_{2}\right)\Theta_{2}}\right\} \end{array}$$

where $\sigma_{yy}^{Tip}=\left\{1+(\beta_{1}+\beta_{2})\frac{a}{b}\right\}\sigma_{yy}^{g}$ is the maximum stress occurring at the notch tip, whereas:

(24) $$\begin{array}{l} r_{i}=\sqrt[{4}]{4x^{2}y^{2}\beta_{i}^{2}+{\left(a^{2}-x^{2}+\left(y^{2}-b^{2}\right)\beta_{i}^{2}\right)}^{2}}\\ \theta_{i}=Arg\left\{\left[\left(x^{2}-a^{2}\right)-\beta_{i}^{2}\left(y^{2}-b^{2}\right)\right]+i\left[-2xy\beta_{i}^{}\right]\right\} \end{array}$$

(25) $$\begin{array}{l} \Theta_{i}=x^{2}+r_{i}{}^{2}+y^{2}\beta_{i}^{2}+2r_{i}\left(x\cos\frac{\theta_{i}}{2}+y\beta_{i}\sin\frac{\theta_{i}}{2}\right)\\ \Lambda_{i}=x\sin\frac{\theta_{i}}{2}+r_{i}\sin\theta_{i}+y\beta_{i}\cos\frac{\theta_{i}}{2}\\ \Omega_{i}=x\cos\frac{\theta_{i}}{2}+r_{i}\cos\theta_{i}-y\beta_{i}\sin\frac{\theta_{i}}{2} \end{array}$$

### 3.2. Mode 2 Loadings

Different from before, the pure mode 2 problem (plate subjected to in-plane shear), can be tackled by taking advantage of the following complex functions:

(26) $$\begin{array}{l} \varphi_{1}=-\frac{i\tau_{xy}^{g}\left(a+b\beta_{1}\right)\left(a+b\beta_{2}\right)}{2\left(z_{1}+\sqrt{z_{1}{}^{2}-a^{2}+b^{2}\beta_{1}^{2}}\right)\left(\beta_{1}-\beta_{2}\right)}\\ \varphi_{2}=-\frac{i\tau_{xy}^{g}\left\{\beta_{2}\left(a\sqrt{z_{2}{}^{2}-a^{2}+b^{2}\beta_{2}^{2}}-bz_{2}\beta_{2}\right)+\beta_{1}\left[az_{2}+b\beta_{2}\left(\sqrt{z_{2}{}^{2}-a^{2}+b^{2}\beta_{2}^{2}}-2z_{2}\right)\right]\right\}}{2\left(\beta_{1}-\beta_{2}\right)\beta_{2}\left(a-b\beta_{2}\right)} \end{array}$$

where z_1_ = x + μ_1_y, z_2_ = x + μ_2_y, and $\tau_{xy}^{g}$ is the far applied shear stress. 

Substituting Equation (26) into Equation (2) gives the stress fields in the form:

(27) $$\begin{array}{c} \sigma_{\mathrm{xx}}=\frac{\tau_{\mathrm{xy}}^{\mathrm{Max}}}{\omega }\cdot \frac{\left(a+b\beta_{1}\right)\left(a+b\beta_{2}\right)\left(r_{1}\beta_{2}^{2}\Lambda_{2}\Theta_{1}-r_{2}\beta_{1}^{2}\Lambda_{1}\Theta_{2}\right)}{r_{1}r_{2}\left(\beta_{1}-\beta_{2}\right)\Theta_{1}\Theta_{2}}\\ \sigma_{\mathrm{yy}}=\frac{\tau_{\mathrm{xy}}^{\mathrm{Max}}}{\omega }\cdot \frac{\left(a+b\beta_{1}\right)\left(a+b\beta_{2}\right)\left(r_{2}\Lambda_{1}\Theta_{2}-r_{1}\Lambda_{2}\Theta_{1}\right)}{r_{1}r_{2}\left(\beta_{1}-\beta_{2}\right)\Theta_{1}\Theta_{2}}\\ \tau_{\mathrm{xy}}=\frac{\tau_{\mathrm{xy}}^{\mathrm{Max}}}{\omega }\left\{1+\frac{\left(a+b\beta_{1}\right)\left(a+b\beta_{2}\right)\left(r_{2}\beta_{1}\Theta_{1}\Omega_{2}-r_{1}\beta_{2}\Theta_{2}\Omega_{1}\right)}{r_{1}r_{2}\left(\beta_{1}-\beta_{2}\right)\Theta_{1}\Theta_{2}}\right\} \end{array}$$

where r_i_, θ_i_, Θ_i_, Λ_i_, and Ω_i_ are defined in Equations (24) and (25), and $\tau_{xy}^{Max}$ is the maximum shear stress occurring along the hole bisector line at a certain distance, x_Max_-a, from the notch tip. Moreover:

(28) $$\omega =\frac{{\overset{⌢}{r}}_{2}\beta_{1}\left(a+b\beta_{1}\right)\left(a+b\beta_{2}\right){\overset{⌢}{\Omega }}_{1}{\overset{⌢}{\Theta }}_{2}+{\overset{⌢}{r}}_{1}{\overset{⌢}{\Theta }}_{1}\left\{{\overset{⌢}{r}}_{2}\left(\beta_{1}-\beta_{2}\right){\overset{⌢}{\Theta }}_{2}-\left[\left(a+b\beta_{1}\right)\beta_{2}\left(a+b\beta_{2}\right){\overset{⌢}{\Omega }}_{2}\right]\right\}}{{\overset{⌢}{r}}_{1}{\overset{⌢}{r}}_{2}\left(\beta_{1}-\beta_{2}\right){\overset{⌢}{\Theta }}_{1}{\overset{⌢}{\Theta }}_{2}}$$

(29) $${\overset{⌢}{r}}_{i}=r_{i}\left[x_{Max},0\right]\;\;\;\;\;{\overset{⌢}{\Theta }}_{i}=\Theta_{i}\left[x_{Max},0\right]\;\;\;\;{\overset{⌢}{\Omega }}_{i}=\Omega_{i}\left[x_{Max},0\right]\;$$

### 3.3. Mode 3 Loadings

Eventually, the pure mode 3 problem (out-of-plane stress fields) can be derived by taking advantage of the following complex function [48]:

(30) $$\varphi_{3}^{}(z_{3})=-\frac{\tau_{zy}^{g}}{k-\beta_{3}(1-k)}\left\{k\sqrt{z_{3}^{2}-a^{2}+\beta_{3}^{2}b^{2}}-\beta_{3}(1-k)z_{3}^{}\right\}$$

where $k=a/\left(a+b\right)$, and the case of k tending to 1 represents the sharp crack case of length 2a, whereas for k tending to 0.5, a circular hole notch can be obtained. 

Accordingly, substituting Equation (30) into Equation (20) allows the stress components to be written as:

(31) τzy=−Reφ3′(z3)=τzygk−β3(1−k)k⋅r3r31⋅r32cosθ3−θ31+θ322−β3(1−k)

(32) τzx=Reμ3φ3′(z3)=β3τzygk−β3(1−k)k⋅r3r31⋅r32sinθ3−θ31+θ322

where:

(33) $$z_{3}=x+i\beta_{3}y=r_{3}e^{i\theta_{3}}\;\;z_{3}^{2}-a^{2}+\beta_{3}^{2}b^{2}=z_{3}^{2}-{\hat{c}}^{2}=\left(z_{3}^{}-{\hat{c}}^{}\right)\left(z_{3}^{}+{\hat{c}}^{}\right)$$

(34) $$\left(z_{3}^{}-{\hat{c}}^{}\right)=r_{31}e^{i\theta_{31}}\;\;\left(z_{3}^{}+{\hat{c}}^{}\right)=r_{32}e^{i\theta_{32}}$$

Stress components can also be re-written, invoking the maximum shear stress at the notch tip, $\tau_{zy}^{tip}=\tau_{zy}^{g}\left(1+\frac{1}{\beta_{3}^{}}\sqrt{\frac{a}{\rho }}\right)$:

(35) $$\begin{array}{l} \tau_{zy}=\tau_{zy}^{tip}\frac{\beta_{3}^{}\sqrt{\frac{\rho }{a}}}{1-\beta_{3}^{2}\frac{\rho }{a}}\left\{\frac{r_{3}}{\sqrt{r_{31}\cdot r_{32}}}\cos\left(\theta_{3}-\frac{\theta_{31}+\theta_{32}}{2}\right)-\beta_{3}\sqrt{\frac{\rho }{a}}\right\}\\ \tau_{zx}=\tau_{zy}^{tip}\frac{\beta_{3}^{2}\sqrt{\frac{\rho }{a}}}{1-\beta_{3}^{2}\frac{\rho }{a}}\left\{\frac{r_{3}}{\sqrt{r_{31}\cdot r_{32}}}\sin\left(\theta_{3}-\frac{\theta_{31}+\theta_{32}}{2}\right)\right\} \end{array}$$

where ρ = b^2^/a.

Along the notch bisector line, Equation (35) simplifies to give:

(36) $$\tau_{zy}=\tau_{zy}^{tip}\frac{\beta_{3}^{}\sqrt{\frac{\rho }{a}}}{1-\beta_{3}^{2}\frac{\rho }{a}}\left\{\frac{\frac{x}{a}}{\sqrt{{\left(\frac{x}{a}\right)}_{}^{2}-1+\beta_{3}^{2}\frac{\rho }{a}}}-\beta_{3}\sqrt{\frac{\rho }{a}}\right\}$$

## 4. Stress Fields for an Orthotropic Finite Plate with Two Symmetric Hyperbolic Notches

### 4.1. Mode 1 Loadings

**Figure 2:**
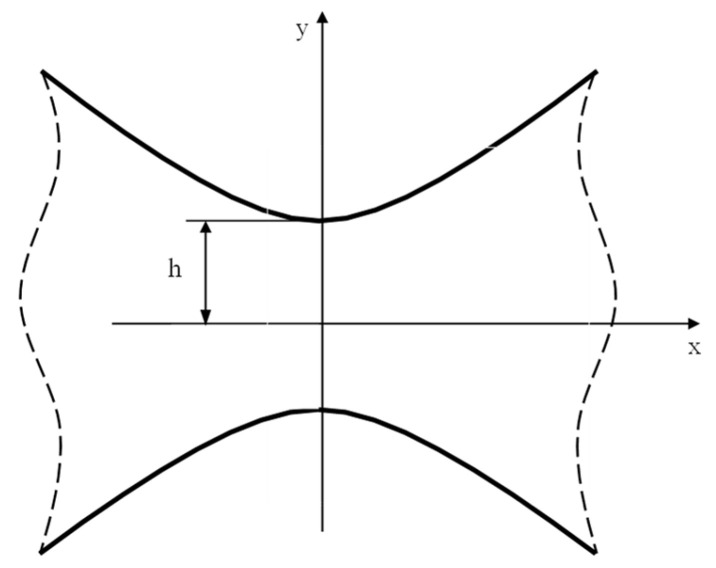
Symmetric hyperbolic notches in an infinite solid and reference system used for Equations (40) and (45).

Consider a plate weakened by two symmetrical hyperbolic notches (Figure 2), which can be obtained by invoking the following complex mapping [49]:

(37) z=c⋅coshξ

where z = x + iy, ξ = u + iv_0_*,* and c is a constant. The case v_0_ = 0 represents the deep crack case whereas, more generally, such a mapping allows two symmetric hyperbolic notches with foci at x=±c to be described, with $h=c\cdot \sin v_{0}$ and $\rho =c\cdot \cot v_{0}\cdot \cos v_{0}$.

The pure mode 1 stress problem (tension applied to the plate) can be determined using the following complex functions [16,45]:

(38) $$\begin{array}{l} \varphi_{1}^{}(z_{1})=-\frac{\sigma_{xx}^{g}}{2}\frac{h}{g}\times Ln\left\{z_{1}+\sqrt{z_{1}^{2}+b^{2}-\mu_{1}^{2}h^{2}}\right\}\\ \varphi_{2}^{}(z_{2})=\frac{\sigma_{xx}^{g}}{2}\frac{h}{g}\times Ln\left\{z_{2}+\sqrt{z_{2}^{2}+b^{2}-\mu_{2}^{2}h^{2}}\right\} \end{array}$$

where:

(39) $$g=\beta_{1}\mathrm{Arctan}\beta_{1}\frac{h}{\rho }-\beta_{2}\mathrm{Arctan}\beta_{2}\frac{h}{\rho }\;\;\;b=\sqrt{\rho \cdot h}$$

$\sigma_{xx}^{g}$ is the nominal stress on the net ligament, and ρ is the root radius at the notch tip.

Substituting Equation (38) into Equation (2) results in:

(40) $$\begin{array}{l} \sigma_{xx}=\sigma_{xx}^{tip}\frac{\sqrt{\rho h}}{\beta_{1}^{2}-\beta_{2}^{2}}\left\{\beta_{1}^{2}{\hat{\rho }}_{1}^{-1/2}\cos\frac{{\hat{\theta }}_{1}}{2}-\beta_{2}^{2}{\hat{\rho }}_{2}^{-1/2}\cos\frac{{\hat{\theta }}_{2}}{2}\right\}\\ \sigma_{yy}=\sigma_{xx}^{tip}\frac{\sqrt{\rho h}}{\beta_{1}^{2}-\beta_{2}^{2}}\left\{{\hat{\rho }}_{2}^{-1/2}\cos\frac{{\hat{\theta }}_{2}}{2}-{\hat{\rho }}_{1}^{-1/2}\cos\frac{{\hat{\theta }}_{1}}{2}\right\}\\ \tau_{xy}=\sigma_{xx}^{tip}\frac{\sqrt{\rho h}}{\beta_{1}^{2}-\beta_{2}^{2}}\left\{\beta_{1}^{}{\hat{\rho }}_{1}^{-1/2}\sin\frac{{\hat{\theta }}_{1}}{2}-\beta_{2}^{}{\hat{\rho }}_{2}^{-1/2}\sin\frac{{\hat{\theta }}_{2}}{2}\right\} \end{array}$$

where:

(41) $${\hat{\rho }}_{j}=\sqrt{{\hat{x}}_{j}^{2}+{\hat{y}}_{j}^{2}}\;\;\;\;\;\;{\hat{\theta }}_{j}=Arg\left\{{\hat{x}}_{j}^{}+i{\hat{y}}_{j}^{}\right\}$$

(42) $${\hat{x}}_{j}^{}=x_{}^{2}-\beta_{j}^{2}y_{}^{2}+\rho h+h^{2}\beta_{j}^{2}\;\;{\hat{y}}_{j}=2\beta_{j}x_{}^{}y_{}$$

Moreover:

(43) $$\sigma_{xx}^{tip}=\sigma_{xx}^{g}\cdot \frac{\beta_{1}^{2}-\beta_{2}^{2}}{g}\sqrt{\frac{h}{\rho }}$$

is the maximum normal stress at the notch tip (x = 0, y = h). 

Along the notch bisector line, x = 0, so that ${\hat{y}}_{j}={\hat{\theta }}_{j}=0$ and:

(44) $${\hat{x}}_{j}^{}=-\beta_{j}^{2}y_{}^{2}+\rho h+h^{2}\beta_{j}^{2}\;\;\;\;{\hat{\rho }}_{j}={\hat{x}}_{j}^{}=-\beta_{j}^{2}y_{}^{2}+\rho h+h^{2}\beta_{j}^{2}$$

Accordingly, the normal stresses become:

(45) $$\begin{array}{l} \sigma_{xx}=\sigma_{xx}^{tip}\frac{\sqrt{h\rho }}{\beta_{1}^{2}-\beta_{2}^{2}}\left\{\frac{\beta_{1}^{2}}{\sqrt{\beta_{1}^{2}{\left(h^{2}-y_{}^{2}\right)}^{{}^{}}+\rho h}}-\frac{\beta_{2}^{2}}{\sqrt{\beta_{2}^{2}{\left(h^{2}-y_{}^{2}\right)}^{{}^{}}+\rho h}}\right\}\\ \sigma_{yy}=\sigma_{xx}^{tip}\frac{\sqrt{\rho h}}{\beta_{1}^{2}-\beta_{2}^{2}}\left\{\frac{1}{\sqrt{\beta_{2}^{2}{\left(h^{2}-y_{}^{2}\right)}^{{}^{}}+\rho h}}-\frac{1}{\sqrt{\beta_{1}^{2}{\left(h^{2}-y_{}^{2}\right)}^{{}^{}}+\rho h}}\right\} \end{array}$$

### 4.2. Mode 2 Loadings

The mode 2 problem can be addressed by taking advantage of the following complex potentials:

(46) $$\begin{array}{l} \varphi_{1}^{}(z_{1})=iA\cdot \beta_{2}Ln\left\{z_{1}+\sqrt{z_{1}^{2}+b^{2}-\mu_{1}^{2}h^{2}}\right\}\\ \varphi_{2}^{}(z_{2})=-iA\cdot \beta_{1}Ln\left\{z_{2}+\sqrt{z_{2}^{2}+b^{2}-\mu_{2}^{2}h^{2}}\right\} \end{array}$$

Substituting Equation (46) into Equation (2) gives:

(47) σxx=A⋅Reiβ2β12z12+ρh+β12h2−iβ1β22z22+ρh+β22h2σyy=A⋅Reiβ2z12+ρh+β12h2−iβ1z22+ρh+β22h2τxy=A⋅Reβ2β1z12+ρh+β12h2−β1β2z22+ρh+β22h2

where parameter A can be linked to the nominal shear stress on the net section, $\tau_{xy}^{n}$, using the following expression:

(48) $$\int_{0}^{h}\tau_{xy}dy=\tau_{xy}^{n}h$$

Substituting Equation (47) into Equation (48) allows one to obtain $A=\tau_{xy}^{n}\frac{h}{\tilde{g}}$, where:

(49) $$\tilde{g}=\left\{\frac{\beta_{2}^{}}{2}Arg\left[i2\beta_{1}\sqrt{\rho /h}+\left(\rho /h-\beta_{1}^{2}\right)\right]-\frac{\beta_{1}^{}}{2}Arg\left[i2\beta_{2}\sqrt{\rho /h}+\left(\rho /h-\beta_{2}^{2}\right)\right]\right\}$$

Eventually, stresses can be re-written taking advantage of the following variables:

(50) $${\hat{\rho }}_{j}=\sqrt{{\hat{x}}_{j}^{2}+{\hat{y}}_{j}^{2}}\;\;\;\;\;\;{\hat{\theta }}_{j}=Arg\left\{{\hat{x}}_{j}^{}+i{\hat{y}}_{j}^{}\right\}$$

(51) $${\hat{x}}_{j}^{}=x_{}^{2}-\beta_{j}^{2}y_{}^{2}+\rho h+h^{2}\beta_{j}^{2}\;\;{\hat{y}}_{j}=2\beta_{j}x_{}^{}y_{}$$

providing:

(52) $$\begin{array}{l} \sigma_{xx}=\tau_{xy}^{\left(n\right)}\frac{h}{\tilde{g}}\left\{\beta_{2}^{}\beta_{1}^{2}{\hat{\rho }}_{1}^{-1/2}\sin\frac{{\hat{\theta }}_{1}}{2}-\beta_{1}^{}\beta_{2}^{2}{\hat{\rho }}_{2}^{-1/2}\sin\frac{{\hat{\theta }}_{2}}{2}\right\}\\ \sigma_{yy}=\tau_{xy}^{\left(n\right)}\frac{h}{\tilde{g}}\left\{\beta_{2}{\hat{\rho }}_{1}^{-1/2}\sin\frac{{\hat{\theta }}_{1}}{2}-\beta_{1}{\hat{\rho }}_{2}^{-1/2}\sin\frac{{\hat{\theta }}_{2}}{2}\right\}\\ \tau_{xy}=\tau_{xy}^{\left(n\right)}\frac{h}{\tilde{g}}\beta_{1}^{}\beta_{2}^{}\left\{{\hat{\rho }}_{1}^{-1/2}\cos\frac{{\hat{\theta }}_{1}}{2}-{\hat{\rho }}_{2}^{-1/2}\cos\frac{{\hat{\theta }}_{2}}{2}\right\} \end{array}$$

### 4.3. Mode 3 Loadings

Whenever the plate is subjected to antiplane shear, the following complex function guarantees that the required boundary conditions are satisfied:

(53) $$\varphi_{3}^{}(z_{3})=-iA\cdot Ln\left\{z_{3}^{}+\sqrt{z_{3}^{2}+\rho \cdot h+\beta_{3}^{2}h^{2}}\right\}$$

Invoking the following auxiliary variables:

(54) $$z_{3}^{2}+\rho \cdot h+\beta_{3}^{2}h^{2}=\left(x^{2}-\beta_{3}^{2}y^{2}+\rho \cdot h+\beta_{3}^{2}h^{2}\right)+i\left(2\beta_{3}^{}xy\right)={\hat{r}}_{3}e^{i{\hat{\theta }}_{3}}$$

and substituting Equation (53) into Equation (20) allows the shear stress components to be determined in closed form as a function of the nominal shear stress on the net section:

(55) $$\begin{array}{l} \tau_{zx}=\frac{2h\tau_{zx}^{n}}{{\mathrm{Arctan}}_{2}\left(\frac{2\beta_{3}\sqrt{\rho /h}}{\rho /h-\beta_{3}^{2}}\right)}\beta_{3}\cdot {\hat{r}}_{3}^{-1/2}\cdot \cos\frac{{\hat{\theta }}_{3}}{2}\\ \tau_{zy}=-\frac{2h\tau_{zx}^{n}}{{\mathrm{Arctan}}_{2}\left(\frac{2\beta_{3}\sqrt{\rho /h}}{\rho /h-\beta_{3}^{2}}\right)}\cdot {\hat{r}}_{3}^{-1/2}\cdot \sin\frac{{\hat{\theta }}_{3}}{2} \end{array}$$

At the notch tip (x = 0 and y = h), the shear stress results to be:

(56) $${\left.\tau_{zx}\right|}_{\begin{array}{c} x=0\\ y=h \end{array}}=\frac{2h\tau_{zx}^{n}}{{\mathrm{Arctan}}_{2}\left(\frac{2\beta_{3}\sqrt{\rho /h}}{\rho /h-\beta_{3}^{2}}\right)}\frac{\beta_{3}^{}}{\sqrt{\rho \cdot h}}$$

Accordingly, the theoretical stress concentration factor is:

(57) $$K_{tn}=\frac{2\beta_{3}^{}}{{\mathrm{Arctan}}_{2}\left(\frac{2\beta_{3}\sqrt{\rho /}h}{\rho /h-\beta_{3}^{2}}\right)}\sqrt{\frac{h}{\rho }}$$

One should note that in the case of sharply curved notches (ρ/h small), Equation (57) simplifies into:

(58) $$K_{tn}≅\frac{2}{\pi }\beta_{3}^{}\sqrt{\frac{h}{\rho }}$$

The shear stress components can also be re-written as a function of the notch tip stress, $\tau_{zx}^{tip}=\tau_{zx}^{n}K_{tn}$.

(59) $$\begin{array}{l} \tau_{zx}=\tau_{zx}^{tip}\cdot \sqrt{\frac{\rho \cdot h}{{\hat{r}}_{3}^{}}}\cdot \cos\frac{{\hat{\theta }}_{3}}{2}\\ \tau_{zy}=-\frac{\tau_{zx}^{tip}}{\beta_{3}}\cdot \sqrt{\frac{\rho \cdot h}{{\hat{r}}_{3}^{}}}\cdot \sin\frac{{\hat{\theta }}_{3}}{2} \end{array}$$

## 5. Stress Fields for Orthotropic Plate with a Deep Parabolic Notch

### 5.1. Mode 1 Problem

**Figure 3:**
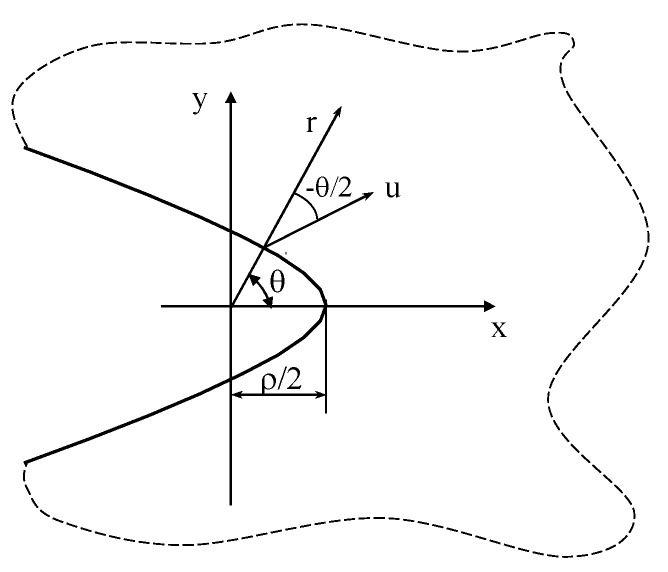
Parabolic notch in an infinite solid and reference system used for Equations (65) and (70).

The problem of an orthotropic plate weakened by a parabolic notch (Figure 3) can be addressed by taking advantage of the mapping function [8]:

(60) $$z=\xi^{2}$$

where z = x + iy and *ξ* = *u*_0_ + i*v*.

The notch apex is at a distance equal to ρ/2 from the origin of the coordinate system, where ρ is the curvature radius at the tip (v = 0).

The mode 1 problem can be addressed using the following complex potentials:

(61) $$\begin{array}{l} \varphi_{1}^{}(z_{1})=A\frac{\mu_{2}^{}}{\mu_{1}^{}-\mu_{2}^{}}z_{1}^{-\frac{1}{2}}\\ \varphi_{2}^{}(z_{2})=-A\frac{\mu_{1}^{}}{\mu_{1}^{}-\mu_{2}^{}}z_{2}^{-\frac{1}{2}} \end{array}$$

In Equation (61), A is a real quantity, while z_j_ are complex variables defined as:

(62) $$z_{j}=\xi_{j}+i\eta_{j}=r_{j}e^{i\theta_{j}}$$

where:

(63) $$\xi_{j}=x^{\prime }+\frac{\rho }{2}\beta_{j}^{2}\;\;\;\eta_{j}=\beta_{j}^{}y^{\prime }$$

Moreover:

(64) $$r_{j}=\sqrt{\xi_{j}^{2}+\eta_{j}^{2}}\;\;\;\theta_{j}=Arg(\xi_{j}+i\eta_{j})$$

In Equations (63) and (64), x′ and y′ are the distances from the notch tip in the x and y directions, respectively. Substituting Equation (61) into Equation (2) gives:

(65) $$\left\{\begin{array}{c} \sigma_{xx}\\ \sigma_{yy}\\ \tau_{xy} \end{array}\right\}=\frac{\sigma_{yy}^{tip}}{\tilde{A}}\left\{\sqrt{\frac{\rho }{r_{1}}}\left(\begin{array}{c} A_{1xx}^{}\cos\frac{\theta_{1}}{2}\\ A_{1yy}^{}\cos\frac{\theta_{1}}{2}\\ -B_{1xy}^{}\sin\frac{\theta_{1}}{2} \end{array}\right)-\sqrt{\frac{\rho }{r_{2}}}\left(\begin{array}{c} A_{2xx}^{}\cos\frac{\theta_{2}}{2}\\ A_{2yy}^{}\cos\frac{\theta_{2}}{2}\\ -B_{2xy}^{}\sin\frac{\theta_{2}}{2} \end{array}\right)\right\}$$

where $\sigma_{yy}^{tip}$ is the maximum notch tip stress and:

(66) $$\tilde{A}=\sqrt{2}\left\{\frac{A_{1yy}^{}}{\beta_{1}^{}}\right.\left.-\frac{A_{2yy}^{}}{\beta_{2}^{}}\right\}\;\;\;A_{1xx}=-\frac{\beta_{1}^{2}\beta_{2}}{{\left(\beta_{1}-\beta_{2}\right)}^{}}\;\;\;A_{2xx}=-\frac{\beta_{2}^{2}\beta_{1}^{}}{{\left(\beta_{1}-\beta_{2}\right)}^{}}$$

(67) $$A_{1yy}=\frac{\beta_{2}}{{\left(\beta_{1}-\beta_{2}\right)}^{}}\;\;\;A_{2yy}=\frac{\beta_{1}}{{\left(\beta_{1}-\beta_{2}\right)}^{}}\;\;\;B_{1xy}=B_{2xy}=\frac{\beta_{1}^{}\beta_{2}^{}}{\beta_{1}-\beta_{2}}$$

Along the notch bisector line (y = 0, x′ > 0):

(68) $$\xi_{j}=x^{\prime }+\beta_{j}^{2}\frac{\rho }{2}\;\;\;\eta_{j}=0$$

(69) $$r_{j}=\xi_{j}^{}=x^{\prime }+\beta_{j}^{2}\frac{\rho }{2}\;\;\;\theta_{j}=0$$

so that the following very simple equation can be found for the normal stress:

(70) $$\frac{\sigma_{yy}}{\sigma_{yy}^{tip}}=\frac{\beta_{1}^{}\;\beta_{2}^{}}{\beta_{1}^{2}-\beta_{2}^{2}}\left[\frac{\beta_{1}^{}\;}{\sqrt{2x^{\prime }/\rho +\beta_{2}^{2}}}-\frac{\beta_{2}^{}\;}{\sqrt{2x^{\prime }/\rho +\beta_{1}^{2}}}\right]$$

### 5.2. Mode 2 Problem

The mode 2 problem can be addressed using the following complex functions:

(71) $$\begin{array}{l} \varphi_{1}^{}(z_{1})=B\frac{1}{\mu_{1}^{}-\mu_{2}^{}}z_{1}^{-\frac{1}{2}}\\ \varphi_{2}^{}(z_{2})=B\frac{1}{\mu_{1}^{}-\mu_{2}^{}}z_{2}^{-\frac{1}{2}} \end{array}$$

where B is a real quantity. Substituting Equation (71) into Equation (2) gives the following stress field:

(72) $$\left\{\begin{array}{c} \sigma_{xx}\\ \sigma_{yy}\\ \tau_{xy} \end{array}\right\}=B\left\{r_{1}^{-1/2}\left(\begin{array}{c} \beta_{1}^{2}\sin\frac{\theta_{1}}{2}\\ -\sin\frac{\theta_{1}}{2}\\ \beta_{1}^{}\cos\frac{\theta_{1}}{2} \end{array}\right)-r_{2}^{-1/2}\left(\begin{array}{c} \beta_{2}^{2}\sin\frac{\theta_{2}}{2}\\ -\sin\frac{\theta_{2}}{2}\\ \beta_{2}^{}\cos\frac{\theta_{2}}{2} \end{array}\right)\right\}$$

where B is a constant to determine, depending on the nominal applied stress, the geometry of the notched body and its elastic properties. 

Along the notch edge, the only non-vanishing stress is:

(73) $$\begin{array}{l} \sigma_{vv}=\frac{\sigma_{xx}+\sigma_{yy}}{2}-\frac{\sigma_{xx}-\sigma_{yy}}{2}\cos\theta -\tau_{xy}\sin\theta =\\ Br_{1}^{-1/2}\left\{\frac{1}{2}\left[\left(\beta_{1}^{2}-1\right)-\cos\theta \left(\beta_{1}^{2}+1\right)\right]\sin\frac{\theta_{1}}{2}-\beta_{1}^{}\cos\frac{\theta_{1}}{2}\sin\theta \right\}\\ -Br_{2}^{-1/2}\left\{\frac{1}{2}\left[\left(\beta_{2}^{2}-1\right)-\cos\theta \left(\beta_{2}^{2}+1\right)\right]\sin\frac{\theta_{2}}{2}-\beta_{2}^{}\cos\frac{\theta_{2}}{2}\sin\theta \right\} \end{array}$$

There are several ways to define parameter B:

1. As a function of the maximum shear stress along the bisector of the notch:
  (74) $$B=\frac{\tau_{xy}^{Max}}{\left\{\frac{\beta_{1}^{}}{\sqrt{2\frac{{\tilde{x}}^{\prime }}{\rho }+\beta_{1}^{2}}}-\frac{\beta_{2}^{}}{\sqrt{2\frac{{\tilde{x}}^{\prime }}{\rho }+\beta_{2}^{2}}}\right\}}=\frac{\tau_{xy}^{Max}}{{\tilde{\tau }}_{xy}^{Max}}$$
  where ${\tilde{x}}^{\prime }$ represents the distance from the notch tip corresponding to the maximum value for the shear stress, τ_xy_.
2. It can be linked to the maximum value of the normal stress, σ_vv_, along the notch boundary:
  (75) $$B=\frac{\sigma_{vv}^{Max}}{{\tilde{\sigma }}_{vv}^{Max}}$$
  where:
  (76) $$\begin{array}{l} {\tilde{\sigma }}_{vv}^{Max}={\tilde{r}}_{1}^{-1/2}\left\{\frac{1}{2}\left[\left(\beta_{1}^{2}-1\right)-\cos\tilde{\theta }\left(\beta_{1}^{2}+1\right)\right]\sin\frac{{\tilde{\theta }}_{1}}{2}-\beta_{1}^{}\cos\frac{{\tilde{\theta }}_{1}}{2}\sin\tilde{\theta }\right\}\\ -{\tilde{r}}_{2}^{-1/2}\left\{\frac{1}{2}\left[\left(\beta_{2}^{2}-1\right)-\cos\tilde{\theta }\left(\beta_{2}^{2}+1\right)\right]\sin\frac{{\tilde{\theta }}_{2}}{2}-\beta_{2}^{}\cos\frac{{\tilde{\theta }}_{2}}{2}\sin\tilde{\theta }\right\} \end{array}$$
  and $\tilde{\theta }$ is the solution of the following equation:
  (77) $${\left.\frac{\partial \sigma_{vv}}{\partial \theta }\right|}_{\begin{array}{c} x^{\prime }=\rho /2\left(\cos^{-2}\frac{\theta }{2}-1\right)\\ y^{\prime }=\rho /2\sin^{-2}\frac{\theta }{2} \end{array}}=0$$
3. It can be linked to a generalized stress intensity factor, K_2ρ_. Indeed, at a proper distance from the notch tip:
  (78) $$B=\frac{1}{\beta_{1}^{}-\beta_{2}^{}}\frac{K_{2\rho }}{\sqrt{\pi \rho }}$$
  where $K_{2\rho }$ is the mode 2 generalized stress intensity factor for the orthotropic blunt crack [50].

### 5.3. Mode 3 Problem

The mode 3 problem associated to a parabolic notch in an orthotropic plate can be addressed by taking advantage of the following complex function:

(79) $$\varphi_{3}^{}(z_{3})=-Bz_{3}^{\frac{1}{2}}$$

where B is a real constant, whereas z_3_ is a complex variable defined as:

(80) $$z_{3}=x_{3}+iy_{3}=r_{3}e^{i\theta_{3}}$$

where:

(81) $$x_{3}=x^{\prime }+\frac{\rho }{2}\beta_{3}^{2}\;\;\;y_{3}=\beta_{3}^{}y^{\prime }$$

(82) $$r_{3}=\sqrt{x_{3}^{2}+y_{3}^{2}}\;\;\;\theta_{3}=Arg(x_{3}+iy_{3})$$

In Equation (81), x′ and y′ are the distances from the notch tip in the x and y directions, respectively.

Stress components can be determined by substituting Equation (79) into Equation (20), leading to the following expressions:

(83) $$\begin{array}{l} \tau_{zy}=\tau_{zy}^{tip}\beta_{3}^{}\sqrt{\frac{\rho }{2r_{3}^{}}}\cos\frac{\theta_{3}}{2}\\ \tau_{zx}=-\tau_{zy}^{tip}\beta_{3}^{2}\sqrt{\frac{\rho }{2r_{3}^{}}}\sin\frac{\theta_{3}}{2} \end{array}$$

where $\tau_{zy}^{tip}$ is the maximum shear stress at the notch tip.

## 6. Lateral Radiused V-Shaped Notches

### 6.1. Mode 1 Loadings

**Figure 4:**
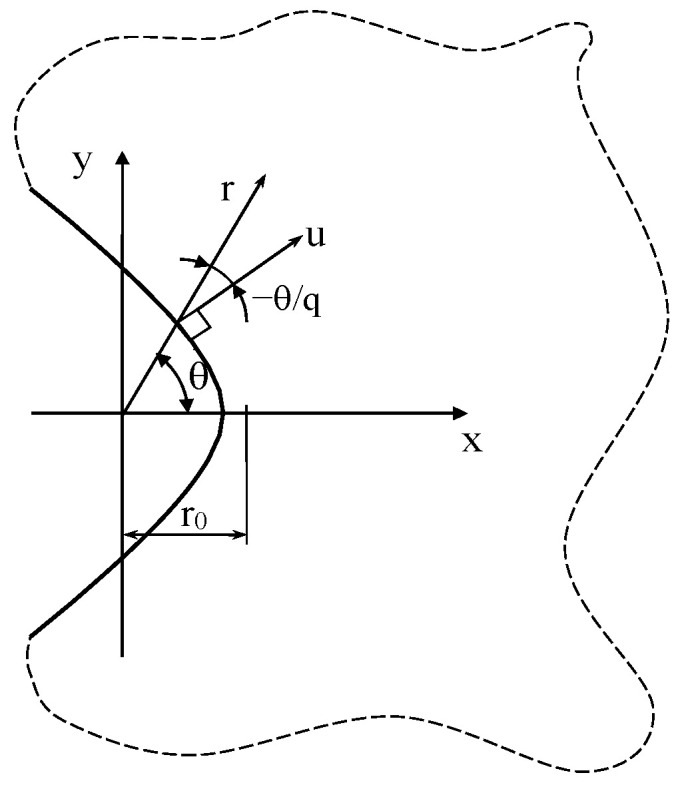
Radiused V-shaped notch in an infinite solid and reference system used for Equations (87)–(89).

The edge of a blunt notch with a generic opening angle and curvature radius at its tip can be described using the following mapping function [8,9] $z=\xi^{q}$ (see also Figure 4), where z=x+iy and  ξ=u+iv, and the notch edge is described by the equation $u=u_{0}$. Moreover:

(84) $$q=\frac{2\pi -2\alpha }{\pi }=\frac{2\gamma }{\pi }\;\;u_{0}={\left(\frac{q-1}{q}\rho \right)}^{\frac{1}{q}}$$

With reference to the coordinate system shown in Figure 4, the solution for this notch problem can be sought using a series formulation for the complex potentials in the form $\varphi_{j}^{\prime }(z_{j})=\sum_{j=1}^{\infty }{\tilde{A}}_{j}z_{j}^{\lambda_{j}-1}$.

However, in order to obtain manageable expressions for the stress fields, the series expansion can be truncated to a finite number of terms, with a tradeoff between the simplicity and accuracy of the associated solution. 

Dealing with the mode 1 problem, Zappalorto and Carraro [45] used a one-term-based solution, obtaining simple yet accurate expressions which were found to be in satisfactory agreement with numerical results, in particular near the notch tip and along the bisector line. 

Some years later, with the aim to improve this last-mentioned solution, and to obtain very accurate stress fields both along and outside of the notch bisector line, Pastrello et al. [46] used the following enriched forms for complex potentials:

(85) $$\varphi_{1}^{\prime }(z_{1})=A_{1}{\left(\frac{z_{1}}{r_{0}}\right)}^{\lambda_{1}-1}+C_{1}{\left(\frac{z_{1}}{r_{0}}\right)}^{\;\;\mu_{1}-1}\varphi_{2}^{\prime }(z_{2})=B_{1}{\left(\frac{z_{2}}{r_{0}}\right)}^{\lambda_{1}-1}+D_{1}{\left(\frac{z_{2}}{r_{0}}\right)}^{\mu_{1}-1}+E_{1}{\left(\frac{z_{2}}{r_{0}}\right)}^{\zeta_{1}-1}$$

where $z_{j}{=x}_{j}{+\mathrm{iy}}_{j}{=r}_{j}e^{{i\theta }_{j}}$ and:

(86) $$x_{j}=x^{\prime }+r_{0}\beta_{j}^{t_{1}}\;\;\;y_{j}=\beta_{j}^{}y^{\prime }\;\;\;r_{j}=\sqrt{\xi_{j}^{2}+\eta_{j}^{2}}\;\;\;\theta_{j}=Arg(\xi_{j}+i\eta_{j})$$

In Equation (86), x′ = x − r_0_ and y′ = y represent the distances from the apex of the notch; instead, A_1_, B_1_, C_1_, D_1_, E_1_, λ_1_, μ_1_, ζ_1_, and t_1_ are real constants to be determined with proper boundary conditions, under the hypothesis that $1<\lambda_{1}<\mu_{1}<\zeta_{1}$. 

Substituting Equation (85) into Equation (9) allows the mode 1 stress components to be explicitly derived:

(87) $$\begin{array}{c} \sigma_{\mathrm{rr}}=\frac{\sigma_{\mathrm{yy}}^{\mathrm{tip}}}{\tilde{A}}\left\{{\left(\frac{r_{1}}{r_{0}}\right)}^{\lambda_{1}-1}\left[k_{11}\cos\left(1-\lambda_{1}\right)\theta_{1}+k_{12}\sin\left(1-\lambda_{1}\right)\theta_{1}\right]+\right.\\ \chi_{12}{\left(\frac{r_{1}}{r_{0}}\right)}^{\mu_{1}-1}\left[k_{11}\cos\left(1-\mu_{1}\right)\theta_{1}+k_{12}\sin\left(1-\mu_{1}\right)\theta_{1}\right]+\\ \chi_{21}{\left(\frac{r_{2}}{r_{0}}\right)}^{\lambda_{1}-1}\left[k_{21}\cos\left(1-\lambda_{1}\right)\theta_{2}+k_{22}\sin\left(1-\lambda_{1}\right)\theta_{2}\right]+\\ \chi_{22}{\left(\frac{r_{2}}{r_{0}}\right)}^{\mu_{1}-1}\left[k_{21}\cos\left(1-\mu_{1}\right)\theta_{2}+k_{22}\sin\left(1-\mu_{1}\right)\theta_{2}\right]+\\ \left.\chi_{23}{\left(\frac{r_{2}}{r_{0}}\right)}^{\zeta_{1}-1}\left[k_{21}\cos\left(1-\zeta_{1}\right)\theta_{2}+k_{22}\sin\left(1-\zeta_{1}\right)\theta_{2}\right]\right\} \end{array}$$

(88) $$\begin{array}{c} \sigma_{\theta \theta }=\frac{\sigma_{\mathrm{yy}}^{\mathrm{tip}}}{\tilde{A}}\left[{\left(\frac{r_{1}}{r_{0}}\right)}^{\lambda_{1}-1}\left[\;m_{11}\cos\left(1-\lambda_{1}\right)\theta_{1}+m_{12}\sin\left(1-\lambda_{1}\right)\theta_{1}\right]+\right.\\ \chi_{12}{\left(\frac{r_{1}}{r_{0}}\right)}^{\mu_{1}-1}\left[\;m_{11}\cos\left(1-\mu_{1}\right)\theta_{1}+m_{12}\sin\left(1-\mu_{1}\right)\theta_{1}\right]+\\ \chi_{21}{\left(\frac{r_{2}}{r_{0}}\right)}^{\lambda_{1}-1}\left[\;m_{21}\cos\left(1-\lambda_{1}\right)\theta_{2}+m_{22}\sin\left(1-\lambda_{1}\right)\theta_{2}\right]+\\ \chi_{22}{\left(\frac{r_{2}}{r_{0}}\right)}^{\mu_{1}-1}\left[\;m_{21}\cos\left(1-\mu_{1}\right)\theta_{2}+m_{22}\sin\left(1-\mu_{1}\right)\theta_{2}\right]+\\ \left.\chi_{23}{\left(\frac{r_{2}}{r_{0}}\right)}^{\zeta_{1}-1}\left[\;m_{21}\cos\left(1-\zeta_{1}\right)\theta_{2}+m_{22}\sin\left(1-\zeta_{1}\right)\theta_{2}\right]\right\} \end{array}$$

(89) $$\begin{array}{c} \tau_{r\theta }=\frac{\sigma_{\mathrm{yy}}^{\mathrm{tip}}}{\tilde{A}}\left\{{\left(\frac{r_{1}}{r_{0}}\right)}^{\lambda_{1}-1}\left[n_{11}\cos\left(1-\lambda_{1}\right)\theta_{1}+n_{12}\sin\left(1-\lambda_{1}\right)\theta_{1}\right]+\right.\\ \chi_{12}{\left(\frac{r_{1}}{r_{0}}\right)}^{\mu_{1}-1}\left[n_{11}\cos\left(1-\mu_{1}\right)\theta_{1}+n_{12}\sin\left(1-\mu_{1}\right)\theta_{1}\right]+\\ \chi_{21}{\left(\frac{r_{2}}{r_{0}}\right)}^{\lambda_{1}-1}\left[n_{21}\cos\left(1-\lambda_{1}\right)\theta_{2}+n_{22}\sin\left(1-\lambda_{1}\right)\theta_{2}\right]+\\ \chi_{22}{\left(\frac{r_{2}}{r_{0}}\right)}^{\mu_{1}-1}\left[n_{21}\cos\left(1-\mu_{1}\right)\theta_{2}+n_{22}\sin\left(1-\mu_{1}\right)\theta_{2}\right]+\\ \left.\chi_{23}{\left(\frac{r_{2}}{r_{0}}\right)}^{\zeta_{1}-1}\left[n_{21}\cos\left(1-\zeta_{1}\right)\theta_{2}+n_{22}\sin\left(1-\zeta_{1}\right)\theta_{2}\right]\right\} \end{array}$$

where:

(90) $$\tilde{A}\;=2\left\{\beta_{1}^{t_{1}\left(\lambda_{1}-1\right)}+\chi_{12}\beta_{1}^{t_{1}\left(\mu_{1}-1\right)}+\chi_{21}\beta_{2}^{t_{1}\left(\lambda_{1}-1\right)}+\chi_{22}\beta_{2}^{t_{1}\left(\mu_{1}-1\right)}+\chi_{23}\beta_{2}^{t_{1}\left(\zeta_{1}-1\right)}\right\}$$

and λ_1_ can be determined by solving the following nonlinear equation [44]:

(91) $$\begin{array}{l} \cos(1-\lambda_{1})\theta_{2}(\gamma )\left\{\cos(1-\lambda_{1})\theta_{1}(\gamma )\right.\left[m_{11}(\gamma )n_{21}(\gamma )-m_{21}(\gamma )n_{11}(\gamma )\right]-\\ \sin(1-\lambda_{1})\theta_{1}(\gamma )\left.\left[m_{21}(\gamma )n_{12}(\gamma )-m_{12}(\gamma )n_{21}(\gamma )\right]\right\}-\\ \sin(1-\lambda_{1})\theta_{2}(\gamma )\left\{\cos(1-\lambda_{1})\theta_{1}(\gamma )\right.\left[m_{22}(\gamma )n_{11}(\gamma )-m_{11}(\gamma )n_{22}(\gamma )\right]-\\ \left.\sin(1-\lambda_{1})\theta_{1}(\gamma )\left[m_{12}(\gamma )n_{22}(\gamma )-m_{22}(\gamma )n_{12}(\gamma )\right]\right\}=0 \end{array}$$

Differently, the remaining seven constants, {t_1_, μ_1_, ζ_1_, $\chi_{12}$,${\;\chi }_{21}$,${\;\chi }_{22}$,${\;\chi }_{23}$}, can be determined by approximating the boundary conditions ${\left.\sigma_{uu}\right|}_{u=u_{0}}={\left.\tau_{uv}\right|}_{u=u_{0}}=0$ (for more details, see [45]). 

### 6.2. Mode 2 Loadings

The mode 2 problem can be addressed using the following complex potentials [47]:

(92) $$\varphi_{1}^{\prime }(z_{1})=-iA_{2}z_{1}^{\lambda_{2}-1}-iC_{2}z_{1}^{\mu_{2}-1}\;\varphi_{2}^{\prime }(z_{2})=-iB_{2}z_{2}^{\lambda_{2}-1}-iD_{2}z_{2}^{\mu_{2}-1}-iE_{2}z_{2}^{\zeta_{2}-1}$$

where $z_{j}{=x}_{j}{+\mathrm{iy}}_{j}{=r}_{j}e^{{i\theta }_{j}}$ and:

(93) $$x_{j}=x^{\prime }+r_{0}\beta_{j}^{t_{2}}\;\;\;y_{j}=\beta_{j}^{}y^{\prime }r_{j}=\sqrt{\xi_{j}^{2}+\eta_{j}^{2}}\;\;\;\theta_{j}=Arg(\xi_{j}+i\eta_{j})$$

In Equation (93), x^′^ = x − r_0_ and y^′^ = y represent the distances from the apex of the notch. A_2_, B_2_, C_2_, D_2_, E_2_, λ_2_, μ_2_, ζ_2_, and t_2_ are real constants to be determined with proper boundary conditions, under the hypothesis that $1<\lambda_{2}<\mu_{2}<\zeta_{2}$. 

Equation (92) provides the following expression for the mode 2 stress field:

(94) $$\begin{array}{c} \sigma_{\mathrm{rr}}=A\left\{{\left(\frac{r_{1}}{r_{0}}\right)}^{\lambda_{2}-1}\left[k_{12}\cos\left(1-\lambda_{2}\right)\theta_{1}-k_{11}\sin\left(1-\lambda_{2}\right)\theta_{1}\right]+\right.\\ +\chi_{12}{\left(\frac{r_{1}}{r_{0}}\right)}^{\mu_{2}-1}\left[k_{12}\cos\left(1-\mu_{2}\right)\theta_{1}-k_{11}\sin\left(1-\mu_{2}\right)\theta_{1}\right]+\\ +\chi_{21}{\left(\frac{r_{2}}{r_{0}}\right)}^{\lambda_{2}-1}\left[k_{22}\cos\left(1-\lambda_{2}\right)\theta_{2}-k_{21}\sin\left(1-\lambda_{2}\right)\theta_{2}\right]+\\ +\chi_{22}{\left(\frac{r_{2}}{r_{0}}\right)}^{\mu_{2}-1}\left[k_{22}\cos\left(1-\mu_{2}\right)\theta_{2}-k_{21}\sin\left(1-\mu_{2}\right)\theta_{2}\right]+\\ \left.+\chi_{23}{\left(\frac{r_{2}}{r_{0}}\right)}^{\zeta_{2}-1}\left[k_{22}\cos\left(1-\zeta_{2}\right)\theta_{2}-k_{21}\sin\left(1-\zeta_{2}\right)\theta_{2}\right]\right\} \end{array}$$

(95) $$\begin{array}{c} \sigma_{\theta \theta }=A\left\{{\left(\frac{r_{1}}{r_{0}}\right)}^{\lambda_{2}-1}\left[\;m_{12}\cos\left(1-\lambda_{2}\right)\theta_{1}-m_{11}\sin\left(1-\lambda_{2}\right)\theta_{1}\right]+\right.\\ +\chi_{12}{\left(\frac{r_{1}}{r_{0}}\right)}^{\mu_{2}-1}\left[\;m_{12}\cos\left(1-\mu_{2}\right)\theta_{1}-m_{11}\sin\left(1-\mu_{2}\right)\theta_{1}\right]+\\ +\chi_{21}{\left(\frac{r_{2}}{r_{0}}\right)}^{\lambda_{2}-1}\left[\;m_{22}\cos\left(1-\lambda_{2}\right)\theta_{2}-m_{21}\sin\left(1-\lambda_{2}\right)\theta_{2}\right]+\\ +\chi_{22}{\left(\frac{r_{2}}{r_{0}}\right)}^{\mu_{2}-1}\left[\;m_{22}\cos\left(1-\mu_{2}\right)\theta_{2}-m_{21}\sin\left(1-\mu_{2}\right)\theta_{2}\right]+\\ \left.+\chi_{23}{\left(\frac{r_{2}}{r_{0}}\right)}^{\zeta_{2}-1}\left[\;m_{22}\cos\left(1-\zeta_{2}\right)\theta_{2}-m_{21}\sin\left(1-\zeta_{2}\right)\theta_{2}\right]\right\} \end{array}$$

(96) $$\begin{array}{c} \tau_{r\theta }=A\left\{{\left(\frac{r_{1}}{r_{0}}\right)}^{\lambda_{2}-1}\left[n_{12}\cos\left(1-\lambda_{2}\right)\theta_{1}-n_{11}\sin\left(1-\lambda_{2}\right)\theta_{1}\right]+\right.\\ +\chi_{12}{\left(\frac{r_{1}}{r_{0}}\right)}^{\mu_{2}-1}\left[n_{12}\cos\left(1-\mu_{2}\right)\theta_{1}-n_{11}\sin\left(1-\mu_{2}\right)\theta_{1}\right]\\ +\chi_{21}{\left(\frac{r_{2}}{r_{0}}\right)}^{\lambda_{2}-1}\left[n_{22}\cos\left(1-\lambda_{2}\right)\theta_{2}-n_{21}\sin\left(1-\lambda_{2}\right)\theta_{2}\right]+\\ +\chi_{22}{\left(\frac{r_{2}}{r_{0}}\right)}^{\mu_{2}-1}\left[n_{22}\cos\left(1-\mu_{2}\right)\theta_{2}-n_{21}\sin\left(1-\mu_{2}\right)\theta_{2}\right]+\\ \left.+\chi_{23}{\left(\frac{r_{2}}{r_{0}}\right)}^{\zeta_{2}-1}\left[n_{22}\cos\left(1-\zeta_{2}\right)\theta_{2}-n_{21}\sin\left(1-\zeta_{2}\right)\theta_{2}\right]\right\} \end{array}$$

Here, λ_2_ is a linear elastic eigenvalue to be determined by solving the following equation:

(97) $$\begin{array}{l} \cos(1-\lambda_{2})\theta_{2}(\gamma )\left\{\cos(1-\lambda_{2})\theta_{1}(\gamma )\right.\left[m_{12}(\gamma )n_{22}(\gamma )-m_{22}(\gamma )n_{12}(\gamma )\right]-\\ \sin(1-\lambda_{2})\theta_{1}(\gamma )\left.\left[m_{11}(\gamma )n_{22}(\gamma )-m_{22}(\gamma )n_{11}(\gamma )\right]\right\}-\\ \sin(1-\lambda_{2})\theta_{2}(\gamma )\left\{\cos(1-\lambda_{2})\theta_{1}(\gamma )\right.\left[m_{21}(\gamma )n_{12}(\gamma )-m_{12}(\gamma )n_{21}(\gamma )\right]-\\ \left.\sin(1-\lambda_{2})\theta_{1}(\gamma )\left[m_{11}(\gamma )n_{21}(\gamma )-m_{21}(\gamma )n_{11}(\gamma )\right]\right\}=0 \end{array}$$

where γ = π − α. Parameters t_2_, μ_2_, ζ_2_, $\chi_{12}$,${\;\chi }_{21}$,${\;\chi }_{22}$,${\;\chi }_{23}$ depend on the notch geometry and material properties and can be determined according to the procedure proposed in ref. [47]. 

As mentioned before for the parabolic notch, the generic parameter A in Equations (93)–(95) can be expressed as a function of the maximum shear stress along the notch bisector, the maximum principal stress along the notch edge, or as a function of a mode 2 Generalized Stress Intensity Factor.

### 6.3. Mode 3 Loadings

In the case of mode 3 loadings, the solution for the stress field can be determined by taking advantage of the following complex potential function:

(98) $$\varphi_{3}^{}(z_{3})=-A_{3}\;z_{3}^{\lambda_{3}}$$

where A_3_ is a real constant, whereas z_3_ is a complex variable defined as:

(99) $$z_{3}=x_{3}+iy_{3}=r_{3}e^{i\theta_{3}}$$

In Equation (98):

(100) $$x_{3}=x^{\prime }+r_{0}\beta_{3}^{t_{3}}\;\;\;y_{3}=\beta_{3}^{}y^{\prime }$$

(101) $$r_{3}=\sqrt{x_{3}^{2}+y_{3}^{2}}\;\;\;\theta_{3}=Arg(x_{3}+iy_{3})$$

and x′ and y′ are the distances from the notch tip in the x and y directions, respectively.

Substituting Equation (97) into Equation (21) allows the shear stress components to be determined:

(102) $$\begin{array}{l} \tau_{z\theta }^{}=\tau_{zy}^{tip}{\left(\frac{r_{0}\;\beta_{3}^{t_{3}}}{r_{3}^{}}\right)}_{}^{1-\lambda_{3}{}^{}}\cdot \left[\cos(1-\lambda_{3}{}^{})\theta_{3}\cos\theta_{}+\beta_{3}\sin(1-\lambda_{3}{}^{})\theta_{3}\sin\theta \right]\\ \tau_{zr}^{}=\tau_{zy}^{tip}{\left(\frac{r_{0}\;\beta_{3}^{t_{3}}}{r_{3}^{}}\right)}_{}^{1-\lambda_{3}{}^{}}\cdot \left[\cos(1-\lambda_{3}{}^{})\theta_{3}\sin\theta_{}-\beta_{3}\sin(1-\lambda_{3}{}^{})\theta_{3}\cos\theta \right] \end{array}$$

where:

(103) $$t_{3}=2-\frac{Ln\frac{q-1}{q(1-\lambda_{3}{}^{})}}{Ln\beta_{3}^{}}$$

and

(104) $$\lambda_{3}=\frac{\pi }{2\theta_{3}(\gamma )}=\frac{\pi }{2\left\{\mathrm{Arctan}\left[\beta_{3}\tan\gamma \right]+\pi \right\}}$$

It is worth noting that in the case of an isotropic material (β_3_ = 1), Equation (101) turns out to be:

(105) $$\begin{array}{l} \tau_{z\theta }^{}=\tau_{z\theta }^{tip}{\left(\frac{r_{0}\;}{r_{}^{}}\right)}_{}^{1-\lambda_{3}{}^{}}\cdot \cos\lambda_{3}\theta_{}\\ \tau_{zr}^{}=\tau_{z\theta }^{tip}{\left(\frac{r_{0}\;}{r_{}^{}}\right)}_{}^{1-\lambda_{3}{}^{}}\cdot \sin\lambda_{3}\theta_{} \end{array}\;\mathrm{with}\;\lambda_{3}=\frac{\pi }{2\gamma }$$

in agreement with the exact solution [13,14,51]. 

## 7. Examples of Application

**Figure 5:**
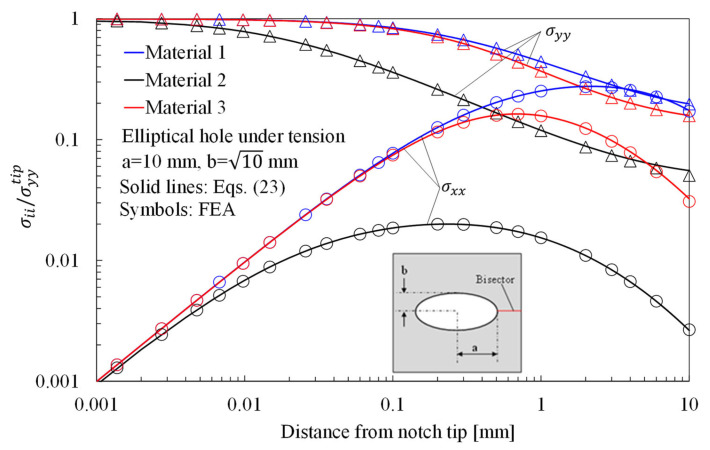
Elliptic hole with a = 10 mm and b = $\sqrt{10}$ mm in a 150 × 150 mm^2^ plate under tension. Mode 1 loadings, different materials. Plot of the normalized stress components $\sigma_{yy}/\sigma_{yy}^{Tip}$ and $\sigma_{xx}/\sigma_{xx}^{Tip}$ along the notch bisector line and comparison with Equation (23). a is the length of the ellipse semi-major axis, while b is the length of the ellipse semi-minor axis.

**Figure 6:**
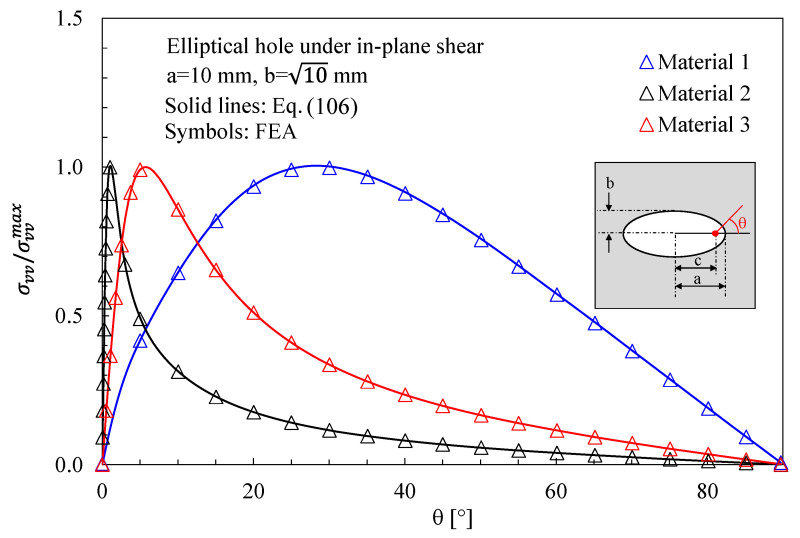
Elliptic hole with a = 10 mm and b = $\sqrt{10}$ mm in a 150 × 150 mm^2^ plate under in-plane shear. Mode 2 loadings, different materials. Plot of the normalized main stress $\sigma_{vv}/\sigma_{vv}^{max}$ along the notch edge and comparison with Equation (106). a is the length of the ellipse semi-major axis, while b is the length of the ellipse semi-minor axis. c is the ellipse linear eccentricity.

**Figure 7:**
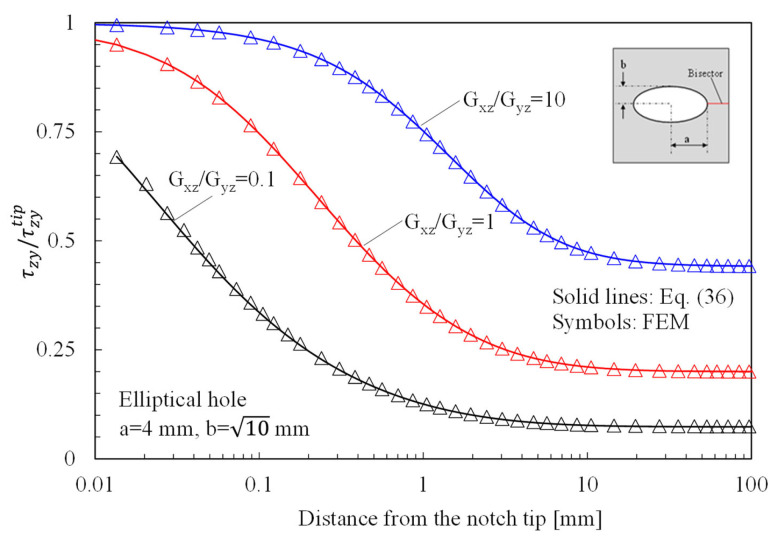
Elliptic hole with a = 4 mm and b = $\sqrt{10}$ mm in a 150 × 150 mm^2^ plate under anti-plane shear. Mode 3 loadings, different materials. Plot of the normalized stress component $\tau_{zy}/\tau_{zy}^{tip}$ along the notch bisector line and comparison with Equation (36). a is the length of the ellipse semi-major axis, while b is the length of the ellipse semi-minor axis.

**Figure 8:**
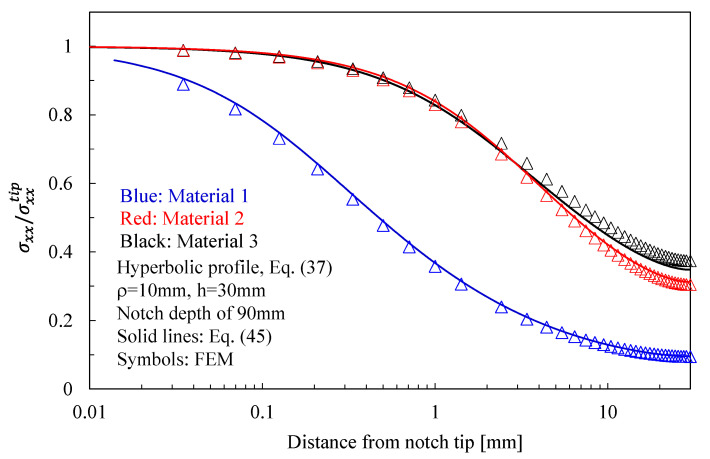
Symmetric hyperbolic notches with ρ = 10 mm, h = 30 mm, and notch depth of 90 mm in a plate with ligament of 60 mm and loaded under tension. Mode 1 loadings, different materials. Plot of the normalized stress component $\sigma_{xx}/\sigma_{xx}^{Tip}$ evaluated along the notch bisector line and comparison with Equation (45). Data adapted from [37].

**Figure 9:**
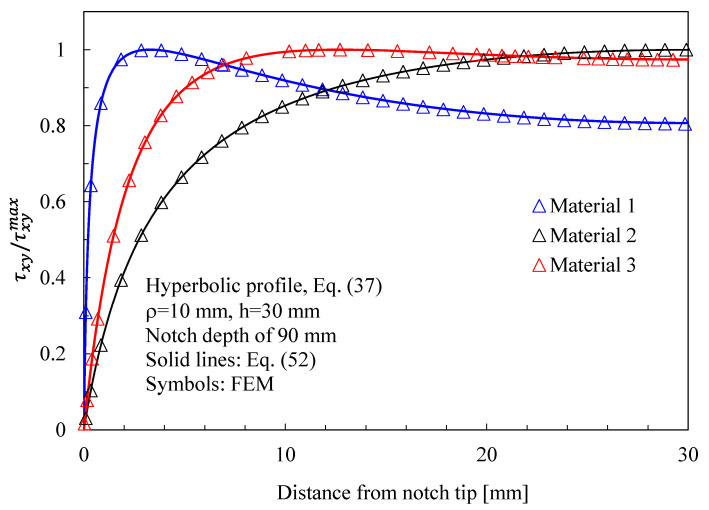
Symmetric hyperbolic notches with ρ = 10 mm, h = 30 mm, and notch depth of 90 mm in a plate with ligament of 60 mm and loaded under in-plane shear. Mode 2 loadings, different materials. Plot of the stress component $\tau_{xy}/\tau_{xy}^{Max}$ along the notch bisector line and comparison with Equation (52). Data adapted from [39].

**Figure 10:**
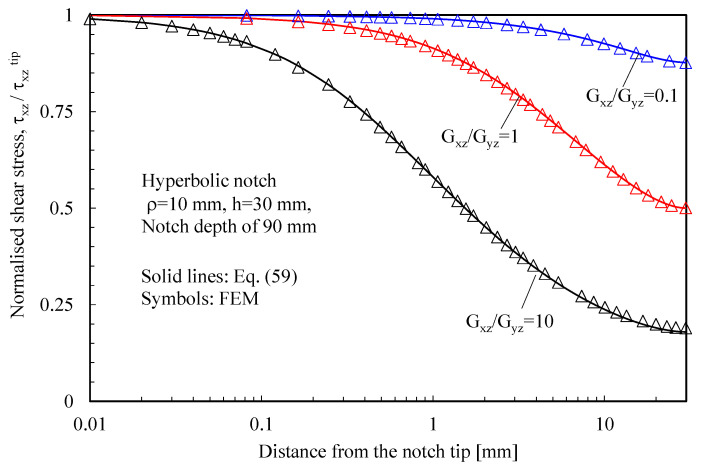
Symmetric hyperbolic notches with ρ = 10 mm and notch depth of 90 mm in a plate with ligament of 60 mm and loaded in anti-plane shear. Mode 3 loadings, different materials. Plot of the stress component $\tau_{xz}/\tau_{xz}^{Tip}$ along the notch bisector line and comparison with Equation (59). Data adapted from [31].

**Figure 11:**
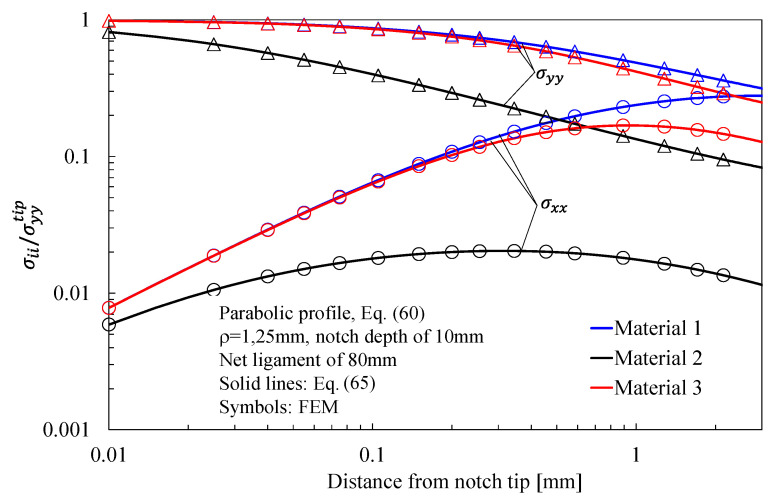
Parabolic notches with ρ = 1.25 mm and notch depth of 10 mm in a plate with ligament of 80 mm and loaded in tension. Mode 1 loadings, different materials. Plot of the normalized stress components $\sigma_{yy}/\sigma_{yy}^{Tip}$ and $\sigma_{xx}/\sigma_{yy}^{Tip}$ along the notch bisector line and comparison with Equation (65). Data adapted from [37].

**Figure 12:**
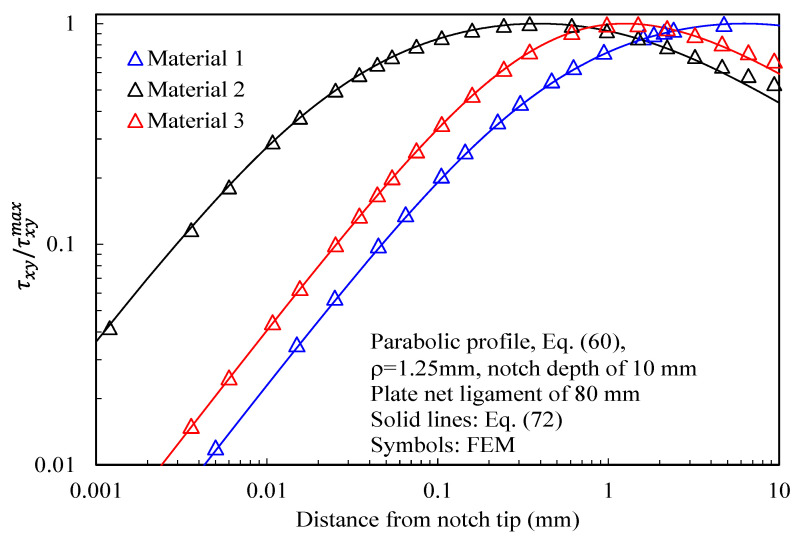
Parabolic notches with ρ = 1.25 mm and notch depth of 10 mm in a plate with ligament of 80 mm and loaded under in-plane shear. Mode 2 loadings, different materials. Plot of the stress component $\tau_{xy}/\tau_{xy}^{Max}$ along the notch bisector line and comparison with Equation (72). Data adapted from [39].

**Figure 13:**
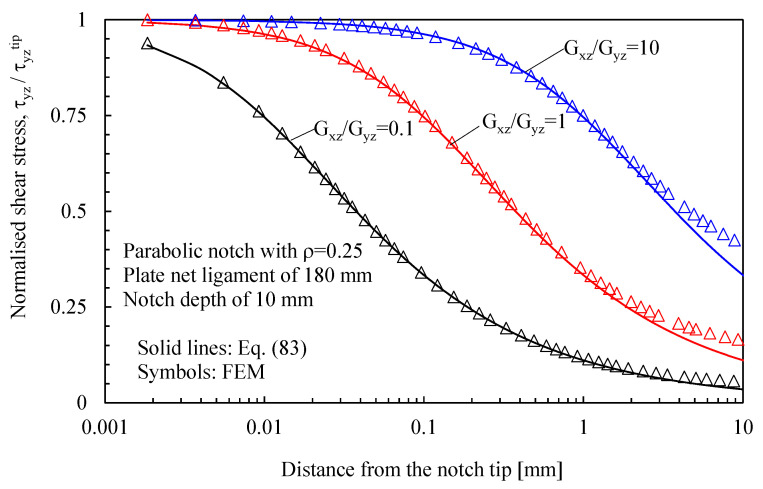
Parabolic notch with ρ = 0.25 mm and depth of 10 mm in a plate with ligament 180 mm and loaded in anti-plane shear. Mode 3 loadings, different materials. Plot of the stress component $\tau_{yz}/\tau_{yz}^{Tip}$ along the notch bisector and comparison with Equation (83). Data adapted from [31].

**Figure 14:**
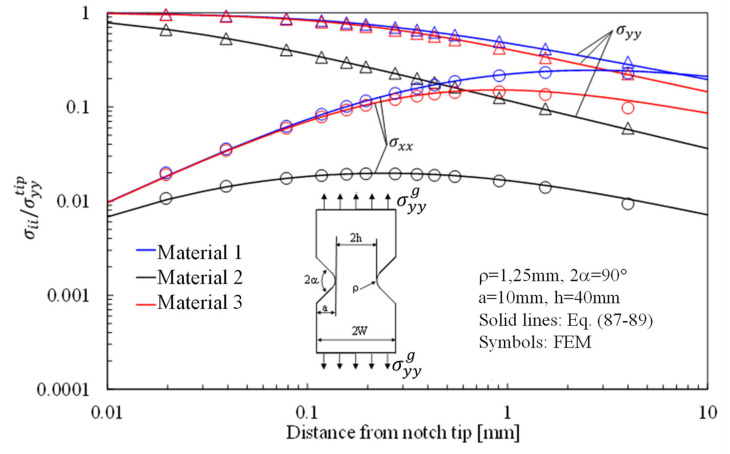
V-shaped blunt notch with ρ = 1 mm, 2α = 90°, and depth of 10 mm in plate with net ligament of 80 mm loaded in tension. Mode 1 loadings, different materials. Plot of the normalized stress components $\sigma_{yy}/\sigma_{yy}^{Tip}$ and $\sigma_{xx}/\sigma_{yy}^{Tip}$ along the notch bisector line and comparison with Equations (87)–(89). Data adapted from [38].

**Figure 15:**
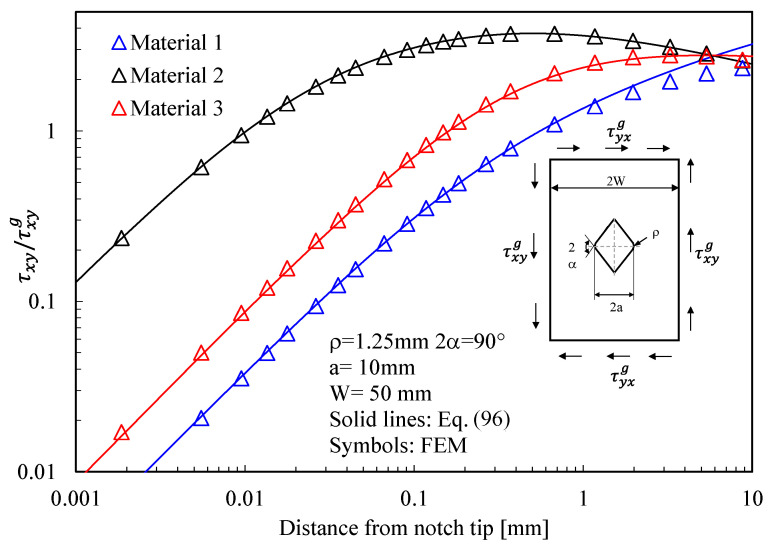
V-shaped radiused hole with ρ = 1.25 mm, 2α = 90°, and depth of 10 mm in a plate with net ligament of 80 mm and loaded under in-plane shear. Mode 2 loadings, different materials. Plot of the stress component $\tau_{xy}/\tau_{}^{Nom}$ along the notch bisector line and comparison with Equation (96). Data adapted from [39].

**Figure 16:**
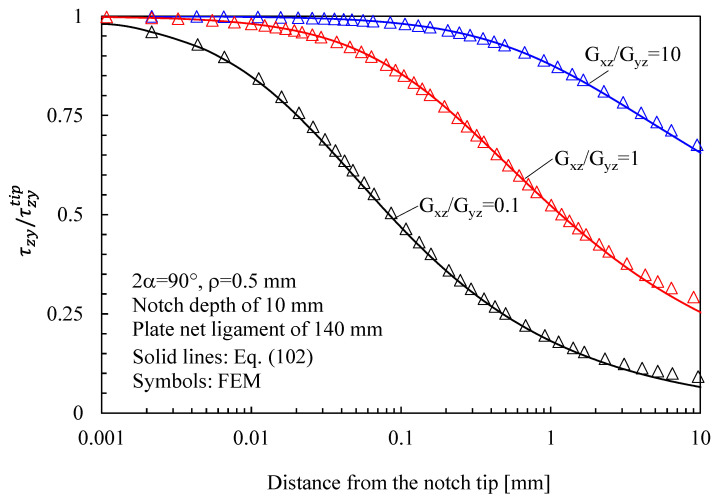
V-shaped notch with ρ = 0.5 mm, 2α = 90° and depth of 10 mm in a plate with net ligament of 140 mm and loaded in anti-plane shear. Mode 3 loadings, different materials. Plot of the normalized stress component $\tau_{zy}/\tau_{zy}^{Tip}$ along the notch bisector line and comparison with Equation (102). Data adapted from [40].

In this section, several examples of application for the solutions described in this paper are presented, considering several materials and different geometries. In particular, Figure 5, Figure 6 and Figure 7 contain new data derived from numerical analyses specifically carried out within this work. Conversely, Figure 8, Figure 9, Figure 10, Figure 11, Figure 12, Figure 13, Figure 14, Figure 15 and Figure 16 contain numerical data taken from the literature, and the original references are reported in their captions.

The results related to mode 1 and mode 2 loadings have been obtained using the following material systems (under the hypothesis that the x-direction corresponds to the notch bisector direction):

1. **Material 1** with the following properties: E_x_ = 160 GPa, E_y_ = 10 GPa, G_xy_ = 5 GPa, ν_xy_ = 0.30 so that β_1_ = 0.7198, β_2_ = 5.5572, representative of a unidirectional Carbon-Fiber Reinforced Epoxy laminate with fibers oriented along the notch bisector line;
2. **Material 2** with the following properties: E_x_ = 10 GPa, E_y_ = 160 GPa, G_xy_ = 5 GPa, ν_xy_ = 0.01875 so that β_1_ = 0.1799, β_2_ = 1.3893, representative of a unidirectional Carbon-Fiber Reinforced Epoxy laminate with fibers oriented along the direction normal to the notch bisector line;
3. **Material 3** with the following properties: E_x_ = 10 GPa, E_y_ = 10 GPa, G_xy_ = 3.846 GPa, ν_xy_ = 0.3 so that β_1_ = 0.9904, β_2_ = 1.0095, representative of a symmetric quasi-isotropic glass fib laminate.

In particular, we have chosen Material 1 and Material 2 since they can be regarded as limiting cases within the context of composites materials, whereas Material 3 has been chosen as an intermediate case between Material 1 and 2.

Differently, several G_xz_/G_yz_ were used to obtain results related to mode 3 loadings. 

The stress distributions in plates with an elliptical hole under tension, in-plane shear and out-of-plane shear are presented in Figure 5, Figure 6 and Figure 7, respectively, considering three different materials. In particular, in Figure 5, the normalized stress components $\sigma_{\mathrm{yy}}/\sigma_{\mathrm{yy}}^{\mathrm{tip}}$ and $\sigma_{\mathrm{xx}}/\sigma_{\mathrm{yy}}^{\mathrm{tip}}$ along the horizontal axis, derived from Equation (23), are compared with the results from the numerical analyses carried out on orthotropic finite size (150 mm × 150 mm) plates under pure tension. As evident, the accuracy of Equation (23) is noteworthy also in the presence of a finite-size solid. 

In Figure 6, the numerical results from finite-size orthotropic plates (150 mm × 150 mm) under shear are compared with the analytical solution reported in Section 3. In this case, the maximum principal stress has been evaluated along the notch edge and compared with the following analytical expression (obtained from Equation (27)):

(106) $$\begin{array}{c} \sigma_{\mathrm{vv}}=\frac{\sigma_{\mathrm{xx}}+\sigma_{\mathrm{yy}}}{2}-\frac{\sigma_{\mathrm{xx}}-\sigma_{\mathrm{yy}}}{2}\cos\theta -\tau_{\mathrm{xy}}\sin\theta =\\ \frac{\tau_{\mathrm{xy}}^{\mathrm{Max}}}{2\omega }\left(\frac{\left(a+b\beta_{1}\right)\left(a+b\beta_{2}\right)\left(r_{1}\left(\beta_{2}^{2}-1\right)\Theta_{1}\Lambda_{2}-r_{2}\left(\beta_{1}^{2}-1\right)\Theta_{2}\Lambda_{1}\right)}{r_{1}r_{2}\left(\beta_{1}-\beta_{2}\right)\Theta_{1}\Theta_{2}}\right)+\\ +\frac{\tau_{\mathrm{xy}}^{\mathrm{Max}}\cos\theta }{2\omega }\left(\frac{\left(a+b\beta_{1}\right)\left(a+b\beta_{2}\right)\left(r_{2}\left(1+\beta_{1}^{2}\right)\Theta_{2}\Lambda_{1}-r_{1}\left(1+\beta_{2}^{2}\right)\Theta_{1}\Lambda_{2}\right)}{r_{1}r_{2}\left(\beta_{1}-\beta_{2}\right)\Theta_{1}\Theta_{2}}\right)+\\ +\frac{\tau_{\mathrm{xy}}^{\mathrm{Max}}\sin\theta }{\omega }\left(\frac{\left(a+b\beta_{1}\right)\left(a+b\beta_{2}\right)\left(r_{1}\beta_{2}\Theta_{2}\Omega_{1}-r_{2}\beta_{1}\Theta_{1}\Omega_{2}\right)}{r_{1}r_{2}\left(\beta_{1}-\beta_{2}\right)\Theta_{1}\Theta_{2}}-1\right) \end{array}$$

In Figure 7, instead, the attention is focused on out-of-plane shear stresses, evaluated along the bisector line of elliptical holes in orthotropic plates subjected to Mode 3 loadings. Numerical results were obtained by considering three different materials ($G_{\mathrm{xz}}/G_{\mathrm{yz}}=0.1;1;10$) and compared with the predictions based on Equation (36). 

The results related to symmetric hyperbolic notches in plates under Mode 1, 2, and 3 loadings are shown in Figure 8, Figure 9 and Figure 10, respectively. Moreover, in this case, the analytical solutions, theoretically valid for infinitely deep notches, are able to describe with great accuracy the numerical results from finite-size solids.

Eventually, the results for blunt V-notches with different notch opening angles are presented in Figure 11, Figure 12, Figure 13, Figure 14, Figure 15 and Figure 16.

In more detail, the results for the stress fields arising in plates with parabolic notches (blunt cracks) under tension, in-plane shear, and out-of-plane shear are summarized in Figure 11, Figure 12 and Figure 13, respectively. Furthermore, for this case, Equations (65), (72) and (83), exact in the case of deep blunt cracks, can be effectively used to characterize the local stress fields arising in finite-size solids. 

Eventually, the attention is moved to lateral radiused notches with a notch opening angle different from zero (2α = 90°), documenting once again the accuracy of the equations proposed in this work (see Figure 14, Figure 15 and Figure 16). 

Based on the results reported in this section, the following main comments can be drawn in relation to the features of the stress fields:

- With regards to Mode 1 loadings, Figure 5, Figure 8, Figure 11 and Figure 14 make it evident that when the material is very stiff along the loading direction (Material 2), the stress gradient is high, and the distribution of the maximum principal stress along the notch bisector line (i.e., the normal stress orthogonal to the notch bisector line) is very steep. Conversely, in the case of a material very stiff along the notch bisector line (Material 1), the stress gradient is much lower, and the distribution of the maximum principal stress along the notch bisector line (i.e., the normal stress orthogonal to the notch bisector line) is mild. As evident, this behavior is general and does not depend on the particular notch geometry under investigation.
- With regards to Mode 2 loadings, from Figure 6, Figure 9, Figure 12 and Figure 15, it is evident that when the material is very stiff along the direction normal to the bisector line (Material 2), the stress gradient is high, and the point, along the notch bisector line, exhibiting the maximum shear stress is very close to the notch tip. This behavior is general and does not depend on the particular notch geometry under investigation.
- Eventually, with reference to Mode 3 loadings, from Figure 7, Figure 10, Figure 13 and Figure 16, it is evident that when G_iz_ is much larger than G_jz_, where j is the direction of the notch bisector line, the stress gradient is high, and the distribution of the maximum antiplane shear stress along the notch bisector line is very steep, independent of the considered notched geometry. Vice versa, when G_iz_ is smaller than G_jz_, the stress gradient is mild.

In order to conclude this section, it is possible to state that the equations and solutions reported in this paper, either exact or approximated, are characterized by a very satisfactory accuracy and represent useful tools to assess the notch stress fields in orthotropic solids weakened by a large variety of geometrical variations.

## 8. Conclusions and Final Remarks

In this work, a brief overview of the analytical solutions available to describe the in-plane and out-of-plane stress fields in orthotropic solids with radiused notches has been presented, and their accuracy discussed versus a number of numerical results. 

In more detail, initially, a brief summary of the fundamentals of complex potentials for orthotropic elasticity was presented, with reference to plane stress or strain and antiplane shear problems.

Subsequently, the attention was moved to the relevant expressions for the notch stress fields, considering elliptical holes, symmetric hyperbolic notches, parabolic notches, blunt cracks, and radiused V-notches. 

Eventually, examples of application were presented, comparing the presented analytical solutions to the results from numerical analyses carried out on relevant cases. 

Based on the cases analyzed, the following main comments can be drawn in relation to the effect of the elastic material properties, independent of the particular notch geometry considered:

- With regards to Mode 1 loadings, when the material is very stiff along the traction direction, the stress gradient is high, and the distribution of the maximum principal stress along the notch bisector line (i.e., the normal stress orthogonal to the notch bisector line) is very steep. Conversely, in the case of a material very stiff along the notch bisector line, the stress gradient is much lower, and the distribution of the maximum principal stress along the notch bisector line is mild.
- With regards to Mode 2 loadings, when the material is very stiff along the direction normal to the bisector line, the stress gradient is high, and the point, along the notch bisector line, exhibiting the maximum shear stress is very close to the notch tip.
- With reference to Mode 3 loadings, when G_iz_ is much larger than G_jz_, where j is the direction of the notch bisector line, the stress gradient is high, and the distribution of the maximum antiplane shear stress along the notch bisector line is very steep. Vice versa, when G_iz_ is smaller than G_jz_, the stress gradient is mild.

As a major conclusion of this work, it can be stated that the equations and solutions reported in this paper, either exact or approximated, are characterized by a very satisfactory accuracy and represent useful tools to assess the notch stress fields in orthotropic solids weakened by a large variety of geometrical variations. 

A final remark concerns the choice of employing an analytical solution or a numerical one (e.g., FEA) in front of a real problem. All the reviewed solutions showed an excellence accuracy when compared with FEA. It means that at an up-front cost of implementing the equations in a spreadsheet or in some programming language (e.g., Python), very accurate solutions at low computational cost could be obtained, saving the cost of running FE simulations.

## Data Availability

The data presented in this study can be retrieved from the referenced papers. If no reference was reported, data are available on request from the corresponding author.
